# Histopathological Assessment of Diseases in Ornamental Fish from 2019 to 2023 in Minas Gerais State, Brazil

**DOI:** 10.3390/ani16203164

**Published:** 2026-10-09

**Authors:** Sóstenes Apolo Correia Marcelino, Leonardo Lima Gorza, Sarah Portes Carneiro, Rafael Romero Nicolino, Hudson Alves Pinto, Guilherme Campos Tavares, Roselene Ecco, Felipe Pierezan

**Affiliations:** 1 Department of Veterinary Clinics and Surgery, School of Veterinary Medicine, Federal University of Minas Gerais (UFMG), Belo Horizonte 31270-901, Minas Gerais, Brazil; sostenesmarcelino@hotmail.com (S.A.C.M.); leonardo_limagorza@hotmail.com (L.L.G.); eccoro.ufmg@gmail.com (R.E.); 2 Department of Preventive Veterinary Medicine, School of Veterinary Medicine, Federal University of Minas Gerais (UFMG), Belo Horizonte 31270-901, Minas Gerais, Brazil; sarahportes7@gmail.com (S.P.C.); rafael.nicolino@gmail.com (R.R.N.); gcamposvet@hotmail.com (G.C.T.); 3 Department of Parasitology, Institute of Biological Sciences, Federal University of Minas Gerais (UFMG), Belo Horizonte 31270-901, Minas Gerais, Brazil; hudsonalvesufmg@gmail.com; 4 Department of Comparative Diagnostic and Population Medicine, College of Veterinary Medicine, University of Florida, Gainesville, FL 32608, USA

**Keywords:** pathology, histopathology, ornamental fish

## Abstract

The present study aimed to investigate the anatomopathological findings in ornamental fish from multiple sources, submitted to the diagnostic service at the Veterinary School of the Universidade Federal de Minas Gerais (UFMG) between 2019 and 2023. Macroscopic and histopathologic examinations were conducted, and the resulting data regarding disease processes were analyzed through a descriptive observational approach. A total of 635 ornamental fish were studied, with a predominance of exotic fish species. The fish were sourced from ornamental fish farms (37.00%; 235/635), shopkeepers (32.44%; 206/635), facilities (18.43%; 117/635), hobbyists (8.66%; 55/635), and wholesalers (3.46%; 22/635). Among the analyzed cases, 50.39% (320/635) presented relevant anatomopathological findings. The main categories of diseases detected were parasitic (56.25%; 180/320), bacterial (20.31%; 65/320), fungal/oomycete (7.50%; 24/320), viral (7.18%; 23/320), neoplastic (1.88%; 6/320), congenital (2.50%; 8/320), and metabolic (0.94%; 3/320). The main parasites found were unidentified digeneans (15.45%; 34/220), presumed *Centrocestus formosanus* (14.55%; 32/220), monogeneans (13.18%; 29/220), presumed *Tetrahymena* spp. (8.18%; 18/220), and presumed *Epistylis* (6.36%; 14/220). There were diagnoses of undocumented diseases in Brazil, such as the suggestive cases of *Ichthyophonus* spp. In addition, novel potential pathogen–host associations in the country were documented, such as *Pleistophora* sp. in *Carnegiella strigata*. This study presents findings that can contribute to determining disease frequency and identifying pathogens based on microscopic lesions.

## Simple Summary

The ornamental fish trade is an expanding economic activity in Brazil and worldwide. Ornamental fish face several sanitary challenges and are susceptible to parasitic, bacterial, fungal, oomycete, viral, neoplastic, and congenital diseases. Therefore, a survey was conducted to determine the frequency of diseases affecting ornamental fish and their potential relationship to the fish’s source by analyzing cases submitted to a diagnostic service in Minas Gerais State, Brazil. All cases were submitted for necropsy and/or histopathology. A total of 635 ornamental fish were examined, primarily sourced from ornamental fish farmers and shopkeepers. The main categories of diseases detected were parasitic and bacterial. Histopathology was an efficient tool in the identification and characterization of various agents, also showing evidence of potential undocumented diseases in Brazil. This study describes findings that can contribute to the identification of agents in lesions, in addition to encouraging new studies that use histopathology in the health of ornamental fish.

## 1. Introduction

Ornamental fish trade is an economic activity in wide expansion in Brazil [1]. In 2022, the trade of ornamental fish in the country surpassed the mark of 20 million specimens and occupied fourth place in the national pet market [2]. An ornamental fish, similar to other aquatic organisms intended for ornamental or aquarium purposes, is defined as any species with a predominantly aquatic habitat, at any stage of development, that is either captured or cultured and primarily kept in aquaria, tanks, or ornamental ponds for aesthetic appreciation, recreation, or educational purposes [1,3]. The most recent Brazilian legislation reports more than 1226 ornamental fish species authorized for use in the domestic market, including 725 native and 501 exotic species [1]. In general, this diversity of fish is included in a chain that involves ornamental fish producers, wholesalers, ornamental fish shopkeepers, and hobbyists [4]. This live animal trade chain occurs with precarious biosecurity conditions and the frequent occurrence of mortality outbreaks caused by various parasitic, bacterial, viral, and fungal agents [5].

In Brazilian ornamental fish trade, several pathogenic agent infections have been reported, including monogenean and digenean trematodes, trichodinids, *Piscinoodinium pillulare*, *Ichthyophthirius multifiliis*, diplomonad flagellates, *Ichthyobodo* sp., *Chilodonella* spp., *Lernaea cyprinacea*, *Tetrahymena* spp., nematodes, cestodes, and branchiurans [6,7]. The most common bacterial diseases include mycobacteriosis [8,9], columnaris disease [5], and infections caused by diverse *Aeromonas* spp. [10]. Reported viral diseases comprise infections by *Lymphocystisvirus* [11,12,13,14], carp edema virus [15], *Cyvirus cyprinidallo* 1 [16], and *CyHV-2* [17], as well as oomycete diseases associated with the genus *Saprolegnia* [5]. Neoplasms such as chromatophoromas [18] are also part of the spectrum of diseases observed in diagnostic routines involving ornamental fish.

Histopathology is a key diagnostic tool for assessing the health status of ornamental fish; however, few studies have investigated fish health using this approach [6,19]. The objective of the present study was to survey the diseases diagnosed in ornamental fish submitted to the Veterinary School of the Federal University of Minas Gerais (UFMG), Brazil, originating from different sources between 2019 and 2023. Histopathology was used as the primary diagnostic method. In addition to describing the main lesions and pathological findings, this study aims to provide histopathological descriptions and photomicrograph images that may assist in identifying pathogenic agents in fish tissues.

## 2. Materials and Methods

### 2.1. Sampling

Retrospective (2019–2021) and prospective (2022–2023) studies were conducted at the Veterinary School of the Federal University of Minas Gerais (UFMG), Brazil, and analyzed all cases submitted to the institution’s diagnostic service during these periods. The retrospective study used data obtained from gross and microscopic findings recorded during routine pathological diagnoses of ornamental fish. For the prospective study, fish specimens were either submitted to the laboratory or collected during field visits in the Zona da Mata mesoregion (Minas Gerais, Brazil), the largest ornamental fish-producing region in the country. Following the collection of moribund and deceased specimens upon demand, we conducted necropsies, histopathological examinations, and systematically tabulated the resulting data. The identification of ornamental fish species in the samples was based on information provided by the suppliers and by comparing the animals’ morphological characteristics with data available in FishBase (https://fishbase.net.br/search.php, accessed on 19 June 2025).

Fish were classified according to their source as originating from hobbyists, facilities, wholesalers, shopkeepers, or fish farmers. In this way, the sampling encompassed different links in the ornamental fish production chain. A hobbyist was defined as an individual who keeps ornamental fish as a hobby and may also reproduce them without commercial purposes. The facility was understood as a site that receives fish from specific sources or conducts its own reproduction, with the main objective of producing healthy fish for experimental use. Wholesalers were defined as establishments that receive fish from producers and whose main activity is the wholesale of fish to retailers. Shopkeepers were considered establishments that receive fish from producers or distributors and primarily sell them to the end consumer (hobbyist). Fish farmers were defined as individuals engaged in the reproduction of ornamental fish for commercial purposes, selling to wholesalers, shopkeepers, or directly to hobbyists.

### 2.2. Necropsy and Histopathology

Most of the cases received consisted of samples fixed in formalin. Live fish received were subjected to euthanasia. Less frequently, euthanasia and necropsy were requested for animals without clinical signs, to assess the health status of fish batches intended for laboratory breeding facilities or commercial producers. Euthanasia was performed by overdose of eugenol (1–3 mL/L) followed by cervical transection, or by rapid cooling for small tropical stenothermal ornamental fish [20], as described and approved by the Animal Ethics Committee (CEUA-UFMG, Protocol 314/2022). This project was registered in SISGEN under code AF01A4F. Necropsies were performed following a standard operating procedure developed by the laboratory. Gross findings were thoroughly described, measured (when applicable), and photographed, considering lesion distribution, size, shape, consistency, and other specific features. Fish up to 5 cm in length were fixed whole, whereas larger fish (up to 5 cm) had their organs removed prior to fixation. Tissue samples were fixed in 10% neutral buffered formalin for 24–48 h at room temperature.

Histopathological processing was performed at the Histopathology Laboratory of the Multidisciplinary Animal Research Unit (MULTILAB, Veterinary School, UFMG, Belo Horizonte, Brazil) following the standard protocol developed by the laboratory. Briefly, tissue samples were decalcified using 24% formic acid for 48–72 h (whole fish, gills, skin, and muscle). Samples were then dehydrated by immersion in a graded ethanol series (70–100%), cleared in xylene, and embedded in paraffin. For whole-fish microtomy, adaptations were made according to the recommendations of the Histopathology Guidance Document for the Medaka Extended One-Generation Reproduction Test [21]. Tissue microsections (4–5 μm) were obtained using a manual rotary microtome (MRP2015, Lupetec, São Carlos, SP, Brazil) and stained with hematoxylin and eosin (H&E) [22]. In addition, Fite-Faraco, Grocott, Periodic Acid–Schiff (PAS), and Giemsa stains were employed to aid in pathogen identification and lesion interpretation [22]. Fish were considered infected whenever pathogens were detected histologically.

### 2.3. Data Compilation and Analysis

Data were collected for each case regarding identification code, year of analysis, common name, family, genus, species, source, morphological diagnoses, affected organs, etiological diagnoses, etiology, type of pathological conclusion, coinfection, special stains, detailed description, and additional observations of the analyzed fish. The information was compiled into Excel spreadsheets, and a descriptive observational study was conducted comparing the frequencies of different diseases among the various species.

In addition, a multiple correspondence analysis (MCA) [23] was performed using the R software v.4.6.1 [24]. The following variables were included in the MCA: fish family, sample origin, and type of disease detected. Variables were considered related when plotted in proximity on the correspondence map.

## 3. Results

### 3.1. Sample Composition, Source and Fish Species

A total of 635 fish were analyzed. Of these, 28 were received in 2019, 31 in 2020, 63 in 2021, 251 in 2022, and 262 in 2023.

Regarding the percentage of fish by source throughout the study period (2019–2023), 37.00% (235/635) of the specimens originated from ornamental fish farmers, 32.44% (206/635) from shopkeepers, 18.43% (117/635) from the animal facility, 8.66% (55/635) from hobbyists, and 3.46% (22/635) from a wholesaler (Table S1).

The samples evaluated comprised fish from 10 orders, 18 families, and 68 different species. Among the specimens analyzed, exotic species predominated (78.27%; 497/635) compared to native ones (21.73%; 138/635) (Table 1). The five most representative families in the dataset were Poeciliidae (23.62%; 150/635), Danionidae (22.50%; 143/635), Osphronemidae (15.28%; 97/635), Cichlidae (9.92%; 63/635), and Cyprinidae (9.76%; 62/635) (Table 1). The main sources of exotic fish were ornamental fish farmers (46.27%; 230/497) and the animal facility (23.54%; 117/497), whereas native fish were provided almost exclusively by shopkeepers (84.78%; 117/138).

In total, 38 exotic species were analyzed. For some fish, identification was possible only at the genus level. The most frequent species were *Danio rerio* (27.16%; 135/497), *Poecilia reticulata* (14.69%; 73/497), *Carassius auratus* (6.84%; 34/497), *Trichogaster trichopterus* (6.63%; 33/497), *Trichogaster lalius* (6.49%; 32/497), and *Betta splendens* (6.04%; 30/497) (Table 1). A total of 30 native species were examined, the most frequent being *Pterophyllum scalare* (16.67%; 23/138), *Carnegiella strigata* (13.76%; 19/138), *Hyphessobrycon eques* (6.52%; 9/138), *Aspidoras pauciradiatus* (5.07%; 7/138), and *Symphysodon discus* (5.07%; 7/138) (Table 1).

### 3.2. Anatomopathological Findings

Among the cases analyzed, 50.39% (320/635) showed some histopathological alteration, while 49.6% (315/635) exhibited no anatomopathological changes (Table S1). Among the defined disease categories, parasitic diseases were the most frequent (56.25%; 180/320), followed by bacteria (20.31%; 65/320), fungal/oomycete (7.50%; 24/320), viral (7.18%; 23/320), neoplastic (1.88%; 6/320), congenital (2.50%; 8/320), and metabolic diseases (0.94%; 3/320). A total of 2.81% (9/320) of the cases presented nonspecific lesions for which it was not possible to determine the etiology and were therefore classified as inconclusive (Table S1).

#### 3.2.1. Parasitic Diseases

A total of 220 fish with parasitic lesions were identified, including cases of primary parasitic diseases and coinfections (Table 2). In 82.72% (182/220) of the cases, the parasitic lesion was considered the main finding, and the final diagnosis was classified as a parasitic disease. In 17.27% (38/220) of the cases, the parasitic lesions were interpreted as secondary or less significant coinfections. Histopathological findings were compatible or suggestive of 19 different parasitic agents.

The main lesions were associated with unidentified digeneans (15.45%; 34/220), presumed *Centrocestus formosanus* (14.55%; 32/220), monogeneans (13.18%; 29/220), presumed *Tetrahymena* spp. (8.18%; 18/220), presumed *Epistylis* (6.36%; 14/220), presumed *Cryptobia* (5.90%; 13/220), capillariids (5.45%; 12/220), *Camallanus* (5.45%; 12/220), presumed *Clinostomum* (5.00%; 11/220), presumed *Trichodina* (4.09%; 9/220), *Eustrongylides* (3.64%; 8/220), presumed *Ichthyophonus* (3.64%; 8/220), and presumed *Piscinoodinium* (3.64%; 8/220) (Table 2).

The main fish species affected by each parasitic disease and their respective origins are presented in Table S2.

##### Trematodes

- Unidentified digeneans

**Figure 1:**
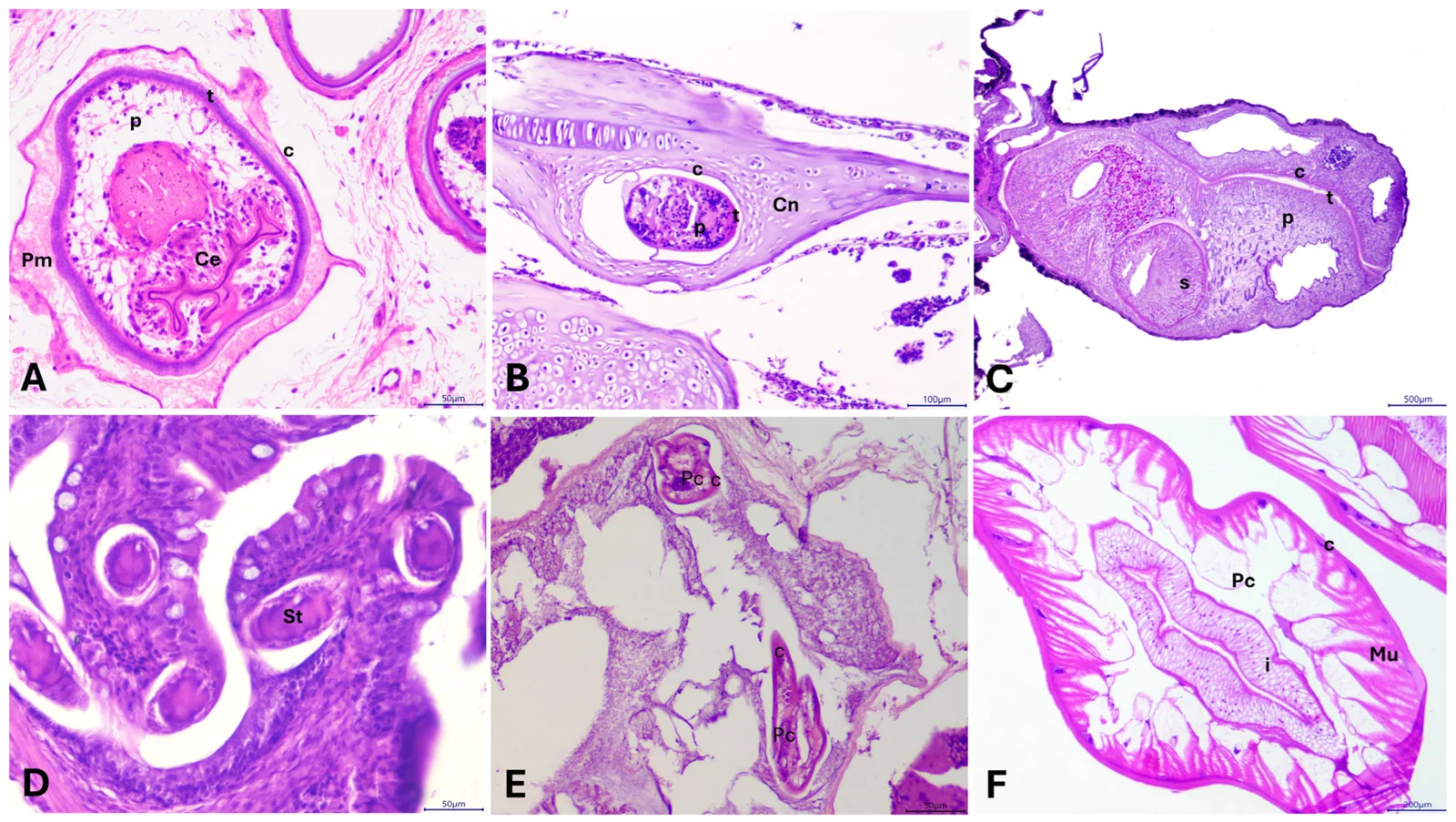
(**A**) Digenean metacercariae in the musculature of *Colomesus asellus*. A capsule (c) and amorphous eosinophilic proteinaceous material (Pm) are observed surrounding the metacercaria, which exhibits a tegument (t), spongy parenchyma (p), and cecum (Ce). H&E stain, 400× magnification. (**B**) Presumed *C. formosanus* metacercaria in the gills of *C. auratus*. Note the cartilaginous nodule (Cn) surrounding the larva, which exhibits a thin capsule (c), tegument (t), and parenchyma (p). H&E stain, 200× magnification. (**C**) Presumed Metacercariae of *Clinostomum* spp. in the skin of *P. reticulata*. Longitudinal section showing the capsule (c) of the cystic structure containing metacercariae, showing a tegument (t), spongy acoelomate parenchyma (p), and a prominent ventral sucker (s). H&E stain, 40× magnification. (**D**) Capillariid nematode in the intestine of *P. reticulata*. Transversal section of an aphasmidian nematode invaginated into the intestinal mucosa, showing a prominent stichosome (St). H&E stain, 400× magnification. (**E**) Nematodes compatible with *Camallanus* spp. in the intestinal mucosa of *P. reticulata*. Note the cuticle (c) and pseudocoelomic cavity (Pc). H&E stain, 400× magnification. (**F**) Transverse sections of an aphasmidian nematode larva compatible with *Eustrongylides* spp. in the coelomic cavity of *Danio rerio*. Note the cuticle (c), coelomyarian–polymyarian musculature (Mu), pseudocoelomic cavity (Pc), and intestine lined by tall columnar cells (i). H&E stain, 100× magnification.

Specimens of different, unidentified species of digenean trematodes were found in 15.45% (34/220) of the fish with parasitic lesions. Digeneans were observed with encysted larval forms (metacercariae) (100–500 µm diameter), surrounded by a thin basophilic wall (2 µm), with a thin eosinophilic tegument, and eosinophilic, “spongy” parenchyma (Figure 1A). In some cases, mild to moderate lymphohistiocytic inflammatory infiltrates were present adjacent to the metacercarial capsule. Occasionally, there was rupture of the cyst wall, accompanied by marked histiocytic inflammation with epithelioid macrophages and multinucleated giant cells.

Digenetic metacercariae were primarily found in *C. strigata* (15/34), *Corydoras* spp. (7/34), and *C. asellus* (3/34) (Table S2). Most of the fish infected with digenetic trematodes originated from shopkeepers (91.18%; 31/34) (Table S1). The location of metacercariae was variable, with the main site being the skeletal musculature (58.82%; 20/34), followed by the liver/hepatopancreas (29.41%; 10/34), coelomic cavity (26.47%; 9/34), skin (20.58%; 7/34), cranial cartilage and bones (14.7%; 5/34), and gills (8.82%; 3/34). In most of the examined fish (76.47%; 27/34), metacercariae were the only pathological finding. However, some fish presented coinfections with presumed: *Saprolegnia* spp. (2/34), *Pleistophora* spp. (3/34), intraluminal myxosporeans in renal tubules (1/34), *I. multifiliis* (1/34), and *Henneguya* sp. (1/34) (Table S1).

- Presumed *Centrocestus formosanus*

Among all specimens presenting parasitic lesions, 14.55% (32/220) were infected with *C. formosanus*. Histologically, lesions associated with the presumed *C. formosanus* infection were characterized by multifocal areas of proliferation of chondroblasts and hypertrophic chondrocytes within the primary gill lamellae, arranged in nodules forming a capsule around the metacercariae (Figure 1B).

Cases of this parasite infection were observed in *D. rerio* (46.82%; 15/32), *C. auratus* (25%; 8/32), and *Xiphophorus hellerii* (15.63%; 5/32) (Table S2). Regarding the source of the infected fish, most specimens were obtained from ornamental fish farmers (84.38%; 27/32) (Table S1). The presumed metacercariae of *C. formosanus* were exclusively located in the gills. In 53.13% (17/32) of the cases, encysted metacercariae represented the only lesion detected. Coinfections were mainly observed with *Eustrongylides* sp. larvae in the coelomic cavity (7/32), monogenean trematodes (3/32), and presumed *Epistylis* spp. (2/32) (Table S1).

- Presumed *Clinostomum* sp.

This parasite was detected in 5% (11/220) of the fish presenting parasitic lesions. Microscopically, transverse and longitudinal sections of encysted metacercariae were observed, surrounded by a basophilic capsule. The metacercariae displayed a thin eosinophilic tegument and a spongy parenchyma containing asucker (Figure 1C).

Presumed *Clinostomum* sp. specimens were found in *P. reticulata* (8/11), all originating from ornamental fish farms, and *P. scalare* (3/11), all obtained from a wholesaler (Table S2). In all cases, the parasite represented the sole pathological finding, located in the skin and musculature (Table S1).

##### Monogeneans

Monogenean were identified in 13.18% (29/220) of the fish presenting parasitic lesions. Microscopically, elongated to oval parasites were observed within the gill interlamellar spaces, with transverse or longitudinal sections (50–100 µm in diameter). The parasites exhibited a thin eosinophilic tegument, an eosinophilic parenchyma, and poorly discernible digestive and reproductive structures. Occasionally, an oral sucker filled with eosinophilic denticles (haptor) could be observed. The histological processing of these parasites often caused parenchymal shrinkage, which made it difficult to visualize internal structures.

The fish species affected by monogeneans were *C. auratus* (15/29), *P. hypophthalmus* (3/29), and *P. reticulata* (3/29) (Table S2). Regarding the source of the infected fish, most specimens were obtained from ornamental fish farms (22/29) (Table S1). Monogenean were exclusively found on the gills. They were identified as the sole pathological finding in 24.14% (7/29) of the cases, or as coinfections in association with presumed *Trichodina* spp. (27.59%; 8/29), presumed *C. formosanus* metacercariae (13.79%; 4/29), gastric granulomas suggestive of *Cryptobia* sp. (10.34%; 3/29), sarcoma (3.45%; 1/29), and granulomatous disease compatible with mycobacteriosis (3.45%; 1/29) (Table S1).

##### Nematodes

- Capillariids

Infection by capillariids accounted for 5.45% (12/220) of the fish presenting parasitic lesions. Microscopically, transverse and longitudinal sections of adult, cylindrical (20–80 µm diameter) aphasmid nematodes, were observed within the intestinal mucosa, were covered by a thin eosinophilic cuticle (2 µm thick), and exhibited a pseudocoelomic cavity, platymyarian musculature, a digestive tube lined by a single layer of cells, bacillary bands, testis, uterus, ovary, and prominent basophilic stichosome (Figure 1D). In some sections, embryonated, bioperculate eggs were visible.

Capillariid infections were detected in *P. reticulata* (6/12), *D. rerio* (5/12), and *C. strigata* (1/12) (Table S2). The main source of the infected fish was research facilities (7/12) and farmers (4/12) (Table S1). In most cases, capillariid infections were the only most important finding (10/12).

- *Camallanus* spp.

Infection by *Camallanus* spp. accounted for 5.45% (12/220) of fish with parasitic lesions. Microscopically, these camallanid nematodes (100–450 µm length/ 50–100 µm diameter) were covered by a thin, smooth, eosinophilic cuticle and exhibited a pseudocoelomic cavity, platymyarian musculature, and tubular digestive and gonadal structures (Figure 1E).

The species in which the parasite was identified included *P. reticulata* (8/12), *P. scalare* (3/12), and *Xiphophorus maculatus* (1/12) (Table S2). Most of the infected fish originated from shopkeepers (83.33%; 10/12) (Table S1). *Camallanus* spp. were observed either as the sole finding (3/12) or in coinfection with presumed systemic tetrahymenosis (5/12) and granulomatous disease suggestive of mycobacteriosis (4/12) (Table S1). The parasites were mostly located in the intestines (9/12) but also occurred in the coelomic cavity (3/12) and near the anus (1/12).

- *Eustrongylides* spp.

Infection compatible with *Eustrongylides* spp. accounted for 5% (8/220) of the parasitic lesions. Histologically, the coelomic cavity was distended and contained transverse and longitudinal sections of larval forms of aphasmid nematodes (1000–2000 µm diameter), covered by a thick eosinophilic cuticle (10–20 µm thick). The larvae exhibited coelomyarian–polymyarian musculature, a pseudocoelomic cavity, and intestinal sections lined by tall columnar epithelial cells (Figure 1F).

All cases of *Eustrongylides* spp. infection were diagnosed in *D. rerio* (8/8) (Table S2). Most of the affected fish originated from ornamental fish farms (87.5%; 7/8), while one case was from a research facility (Table S1).

##### Protozoa

- Presumed *Tetrahymena* spp.

**Figure 2:**
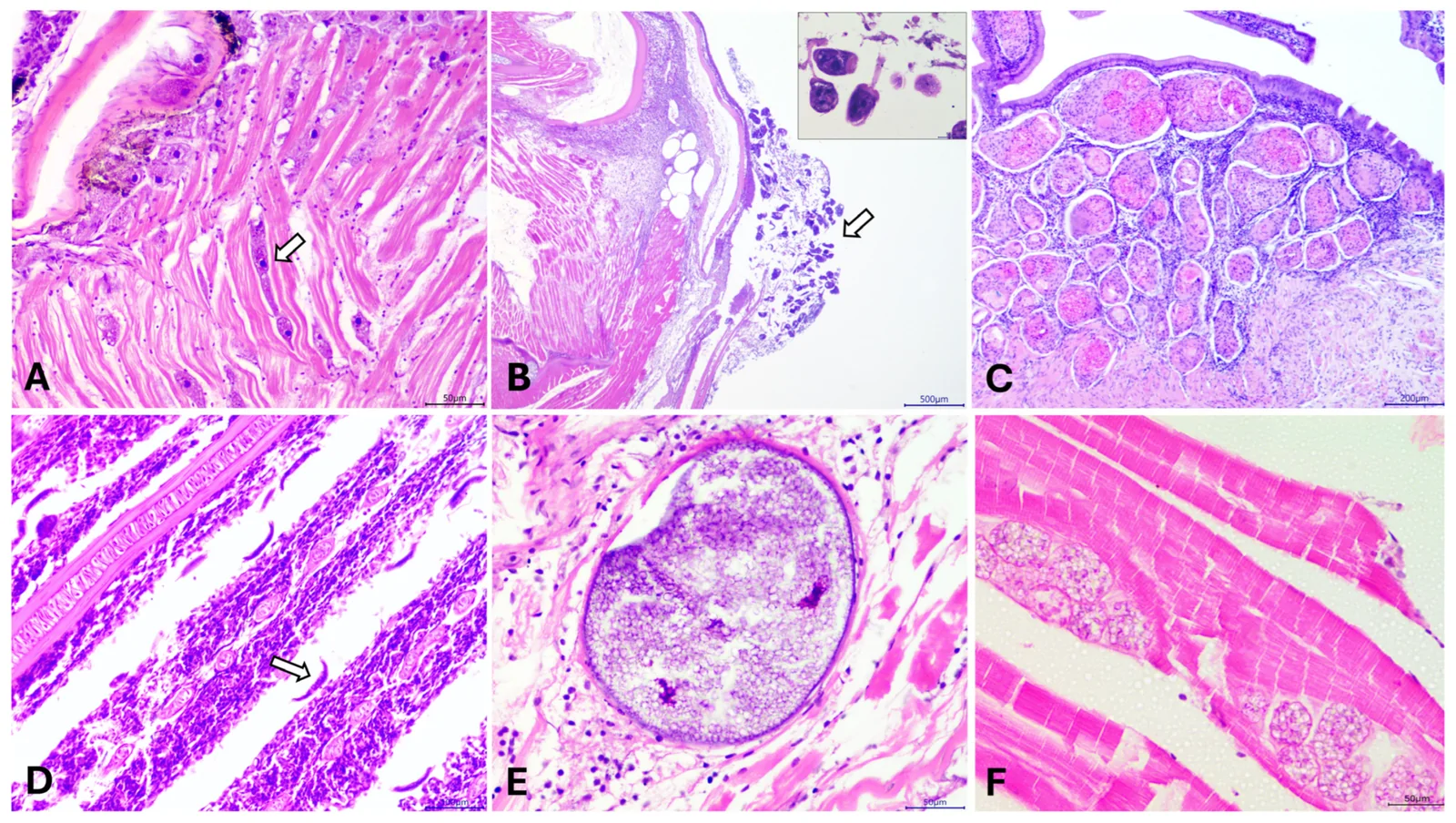
(**A**) Presumed *Tetrahymena* sp. infection in *P. reticulata*. Ciliated trophozoites causing muscular necrosis, minimal inflammatory response and replacement of muscle fibers (arrow). H&E stain, 400× magnification. (**B**) Ulcerative dermatitis on the dorsum of *X. maculatus* with numerous protozoa suggestive of *Epistylis* sp. infection (arrow). H&E stain, 40× magnification. Inset: higher magnification showing zooids supported by a branched stalk. H&E stain, 400× magnification. (**C**) Granulomas in the gastric submucosa of *S. discus* suggestive of *Cryptobia* spp. infection. H&E stain, 100× magnification. (**D**) Presumed *Trichodina* spp. in the gills of *C. auratus*. Longitudinal sections of protozoan organisms (arrow) observed within the interlamellar spaces. H&E stain, 200× magnification. (**E**) Intramuscular cysts suggestive of *Ichthyophonus* sp. in *Danio rerio*. H&E stain, 400× magnification. (**F**) Presumed *Pleistophora* sp. infection in *C. strigata*. Sporoblasts of *Pleistophora* sp. within skeletal muscle fibers. H&E stain, 400× magnification.

Among the parasitic lesions, 8.18% (18/220) of the cases were infections by presumed *Tetrahymena* spp. specimens. Microscopically, these organisms were oval ciliated trophozoites (30–70 µm diameter) with a thin cell wall covered circumferentially by evenly distributed cilia. The cytoplasm was amphophilic and vacuolated and the macronucleus was irregularly oval to elongated and basophilic. The infected tissues were typically necrotic and often exhibited mild or absent inflammatory infiltrates (Figure 2A).

This protozoan organism was found mainly in *P. reticulata* (14/18) (Table S2). The cases originated from shopkeepers (10/18), farms (7/18), and one from a hobbyist (1/18) (Table S1). The protozoan was observed either as a single finding (12/18) or in coinfection compatible with *Camallanus* spp. (5/18). The parasite was primarily located in the dermis (17/18), skeletal musculature (17/18), coelomic cavity (12/18), gills (9/18), periocular adipose tissue (9/18), and oral cavity (5/18). In all cases, organisms were present in multiple sites, and it was frequently detected within blood vessels, underscoring its systemic nature and pathogenic potential.

- Presumed *Epistylis* sp.

Infections caused by this protozoan organism accounted for 6.36% (14/220) of fish with parasitic diseases. Microscopically, transverse or longitudinal sections of conical protozoa were observed, composed of zooids measuring 50–100 µm in diameter, supported by peduncles and attached to the skin (Figure 2B). The zooids were rounded or bell-shaped, featuring rounded or C-shaped macronuclei.

The main fish species infected by these organisms included *Cyprinus carpio* (9/14), *X. maculatus* (2/14), *P. hypophthalmus* (1/14), *R. octofasciata* (1/14), and *T. lalius* (1/14) (Table S2). Most affected fish originated from ornamental fish farms (12/14) (Table S1). In all cases, the protozoan was located on the skin. Secondary bacterial infections were observed in some cases (3/14), as well as coinfections with presumed oomycetes (2/14). In addition, coinfection with metacercariae of presumed *C. formosanus* (2/14) was identified, and granulomatous inflammatory reactions associated with the protozoa were noted in some instances (2/14).

- Flagellates suggestive of *Cryptobia* sp.

Lesions suggestive of *Cryptobia* sp. infection accounted for 5.90% (12/220) of the fish with parasitic diseases. These cases presented nodular, well-defined structures in the submucosa of the stomach and intestines and, occasionally, in the liver and pancreas. The nodules were composed of macrophages and epithelioid macrophages arranged concentrically, forming granulomas. Frequently, the centers of these granulomas contained cellular debris and small, rounded, basophilic structures up to 2 µm in diameter (Figure 2C).

The fish species diagnosed with granulomatous lesions suggestive of *Cryptobia* sp. belonged exclusively to the family Cichlidae, including *Aulonocara* spp. (7/12) and *S. discus* (4/12) (Table S2). Most of the affected fish originated from shopkeepers (8/12), while a smaller number came from farms (4/12) (Table S1).

Regarding lesion distribution, all fish exhibited gastric granulomas (12/12), with some also showing intestinal (3/12) or hepatic granulomas (1/12). A considerable proportion of the cases diagnosed with this possible etiology were coinfected with skin diseases, including ulcerative dermatitis (3/12) or dermatitis caused by oomycete infection (4/12) (Table S1).

- Presumed *Trichodina* spp.

Among the fish with parasitic diseases, 4.09% (9/220) were attributed to presumed *Trichodina* spp. infections. Microscopically, the appearance of this organism varied according to the plane of sectioning. In transverse sections, the protozoa exhibited a discoidal shape, whereas in longitudinal sections they appeared cylindrical. The organisms measured 30–100 µm in diameter and possessed a prominent oral adhesive disc containing characteristic refractile denticles (Figure 2D). Occasionally, a cylindrical or “C”-shaped macronucleus was observed. All cases were diagnosed in *C. auratus*, originating from farms, and were coinfected with monogenean trematodes (Table S1).

- Presumed *Piscinoodinium pillulare*

Cases suggestive of *Piscinoodinium pillulare* infection accounted for 3.64% (8/220) of the fish with parasitic lesions. Microscopically, the protozoa were attached to the gills, olfactory sinuses, oral cavity, or skin, and were characterized by trophonts that were predominantly pyriform, occasionally rounded, measuring 30–60 µm in diameter. They were enclosed by a thin hyaline capsule and exhibited abundant vacuolated cytoplasm with an eosinophilic nucleus. The affected species included *B. splendens* (4/8) and *D. rerio* (3/8) (Table S2). Most cases originated from hobbyists (6/8). Only two cases showed coinfection: one presented granulomatous hepatitis, and another was coinfected with presumed *Ichthyobodo* spp.(Table S1).

- Presumed *Ichthyophthirius multifiliis*

Diagnoses suggestive of *I. multifiliis* infection accounted for only 1.82% (4/220) of the fish which were diagnosed with parasitic diseases. Microscopically, intraepithelial protozoan cysts containing trophonts were noted; these were spherical to ovoid, measuring 100–1000 µm in diameter, covered by short cilia, and surrounded by a hyaline wall. The cytoplasm was amphophilic and vacuolated, containing eosinophilic spherical inclusions and a strongly basophilic macronucleus that was cylindrical in immature trophonts and C-shaped in mature forms. The infection was generally associated with moderate hyperplasia of stratified epithelial and mucus-producing cells in the skin and secondary gill lamellae.

This ciliated protozoan was identified in *B. splendens* (2/4), *C. asellus* (1/4), and *Trichopodus trichopterus* (1/4) (Table S2). All cases originated from wholesalers. Proliferative dermatitis was consistently observed, and one case showed coinfection with oomycete/fungal agents multifocal digenean trematodes, and renal myxosporeans (1/4) (Table S1).

##### Mesomycetozoea

- Presumed *Ichthyophonus* spp.

Cases of presumed *Ichthyophonus* spp. infection accounted for 3.64% (8/220) of the fish which were diagnosed with parasitic diseases. Microscopically, the lesions were characterized by multifocal, round to oval cysts measuring 50–300 μm in diameter, surrounded by a thick basophilic capsule 3–5 μm thick, and filled with numerous oval spores up to 4 μm in diameter, which appeared predominantly as negative (refringent) images and were occasionally faintly basophilic. The cysts were located within the dermis, and among skeletal muscle fibers, and were accompanied by a mild lymphohistiocytic inflammatory infiltrate (Figure 2E). When cyst rupture occurred, they were surrounded by a granulomatous inflammatory response composed of numerous macrophages, epithelioid macrophages, and multinucleated giant cells. This mesomycetozoa was detected in *P. weitzmani* (5/8), *D. rerio* (2/8), and *P. simulans* (1/8) (Table S2). Most cases originated from shopkeepers (6/8), while two were from facilities (2/8) (Table S1). All affected fish exhibited skeletal muscle lesions, which represented the primary pathological finding in each case.

##### Microsporidia

- Presumed *Pleistophora* sp.

Fish with infection suggestive of *Pleistophora* sp. represented 1.36% (3/220) of the parasitic diseases. The microsporidian was characterized by numerous microorganisms at different developmental stages within the sarcoplasm of skeletal muscle fibers. These organisms were predominantly observed as numerous rounded structures, 7–15 µm in diameter, amphophilic with a refractile center (spores), arranged in clusters within cysts measuring 20–50 µm in diameter, surrounded by eosinophilic walls approximately 3 µm thick (sporoblasts). Associated lesions included areas of muscle necrosis and lymphohistiocytic inflammatory infiltrates, particularly evident following sporoblast rupture (Figure 2F). All cases occurred in *C. strigata* originating from shopkeepers and exhibited coinfection with digenetic trematodes (Table S1).

##### Myxosporea

- Suggestive of renal myxosporean

In 1.36% (3/220) of the parasitic disease cases, a cnidarian organism was identified within the renal tubules, suggesting infection by a renal myxosporean. These organisms were represented by oval to polygonal, eosinophilic, were well delineated trophozoites measuring 10–30 µm in diameter. Within the spores, rounded basophilic structures measuring 2–5 µm in diameter (sporoplasms) were observed, occasionally giving the parasite a “rosette-like” appearance. The organisms were mainly located in the lumen of renal tubules, and occasionally dispersed throughout the renal parenchyma. One of these cases occurred in *C. strigata* originating from a shopkeeper, which was also infected with metacercariae of digenetic trematodes. Another case was found in *C. asellus*, which also presented dermatitis caused by oomycete/fungal infection, digenetic trematodes, and *Ichthyophthirius multifiliis* sourced from wholesalers. The last case occurred in *C. auratus* from a farmer and was coinfected with monogenean trematodes (Table S1).

#### 3.2.2. Bacterial Diseases

A total of 66 fish were diagnosed with anatomopathological findings compatible or suggestive of bacterial disease. Additionally, 11 other fish, nine with parasitic diagnoses and two with neoplastic diagnoses, also presented lesions indicative of bacterial infections. Thus, the total number of fish with lesions suggestive of bacterial etiology was 77.

- Compatible with or suggestive of Mycobacteriosis

**Figure 3:**
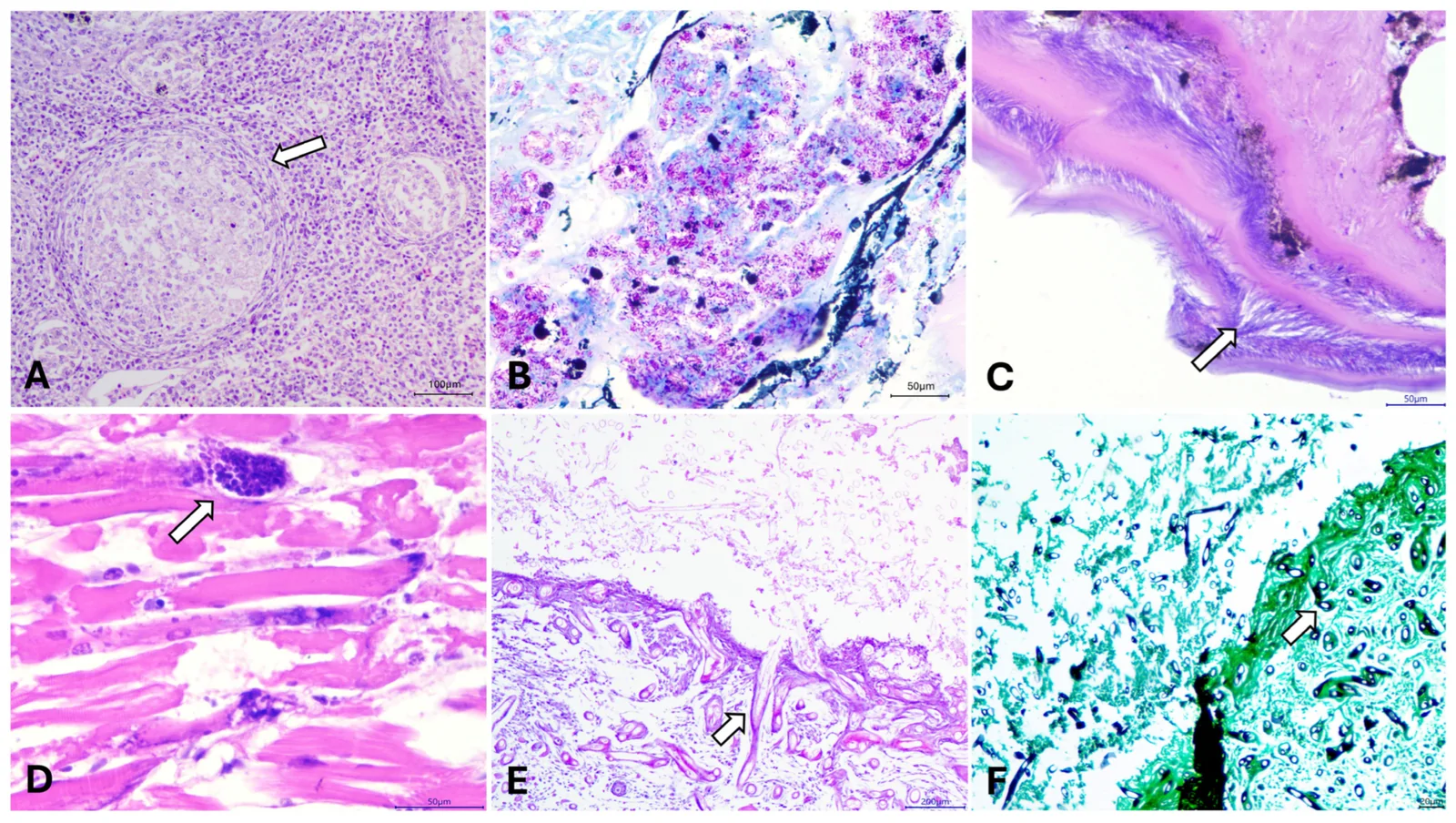
(**A**) Granuloma characterized by a predominance of epithelioid macrophages forming nodular aggregates (arrow) in the kidney of *P. scalare*. H&E stain, 200× magnification. (**B**) Numerous acid-fast bacilli within epithelioid macrophages in the kidney of *Hyphessobrycon amandae*. Fite–Faraco stain, 400× magnification. (**C**) Necrotic caudal fin containing myriad basophilic filamentous bacteria (arrow). H&E stain, 400× magnification. (**D**) Muscular necrosis associated with a mild lymphohistioplasmacytic inflammatory infiltrate (arrow). H&E stain, 600× magnification. (**E**) Water mold infection in *Hemichromis bimaculatus*. Oomycete necrotizing dermatitis with numerous intralesional hyphae (arrow). H&E stain, 100× magnification. (**F**) Silver impregnation of intralesional hyphae (arrow). Grocott’s stain, 200× magnification.

Among the bacterial lesions, 87.01% (67/77) were compatible with or suggestive of mycobacteriosis. Histologically, granulomas were composed of concentric aggregates of macrophages, lymphocytes, pigmented macrophages, and epithelioid macrophages surrounding a core of necrotic material. Some granulomas exhibited an “acute” pattern, characterized byaggregates of numerous epithelioid macrophages with expanded cytoplasm forming nodules encased by a delicate fibrous capsule (“immature granulomas”). Others displayed a more “chronic” appearance, with epithelioid macrophages possessing intensely eosinophilic cytoplasm arranged concentrically around the necrotic center (“mature granulomas”). In many cases, granulomas caused extensive tissue loss and replacement of the normal parenchyma (Figure 3A).

Systemic granulomatous disease consistent with mycobacteriosis was diagnosed in 24 ornamental fish species, with the most frequently affected being *P. scalare* (13/67), *C. auratus* (10/67), and *P. reticulata* (9/67). The affected fish originated from shopkeepers (40/67), farms (12/67), hobbyist (11/67), and wholesalers (4/67) (Table S1). The main organs containing granulomas were the kidney (35/67), liver (24/67), spleen (17/67), and intestine (11/67). Among the 67 cases suggestive of mycobacteriosis, 56 (56/67) were subjected to Fite-Faraco staining for acid-fast bacilli detection, of which 24 (42.86%) tested positive (Figure 3B, Table S1).

- Presumed columnaris disease

Cases suggestive of columnaris disease accounted for 12.99% (10/77) of the bacterial diseases. Microscopically, there were extensive and deep ulcerative lesions associated with numerous filamentous bacteria (20–40 µm in length) morphologically compatible with *Flavobacterium* spp. (Figure 3C). These bacteria were often accompanied by areas of muscle necrosis and mild to absent inflammatory response (Figure 3D).

Suspected cases occurred in *H. eques* (5/10), *D. rerio* (2/10), *P. scalare* (1/10), *Thayeria boehlkei* (1/10), and *T. lalius* (1/10). The affected fish originated from shopkeepers (6/10) and farms (4/10) (Table S1). In most cases, necrotizing dermatitis with intralesional filamentous bacteria was the only lesion observed (9/10). One fish presented coinfection with presumed *C. formosanus* metacercariae and *Eustrongylides* larvae.

#### 3.2.3. Oomycete/Fungal Diseases

A total of 31 cases presented lesions consistent with oomycete/fungal infections. Of these, 23/31 were diagnosed as primary disease, while 8/31 where likely secondary and associated with significant parasitic coinfections (Table S1). Microscopically, they were characterized by numerous filamentous hyphae measuring 1–30 µm in width and 20–150 µm in length, with thin, parallel walls and a negatively stained central lumen. The hyphaeinduced marked necrosis of the skin and skeletal musculature, accompanied by lymphoplasmacytic and histiocytic inflammation with epithelioid macrophages and multinucleated giant cells when the hyphae penetrated deeper muscle layers (Figure 3E,F).

Lesions were recorded in 15 different fish species, mainly *T. lalius* (10/31), *C. asellus* (3/31), *Aulonocara* spp. (3/31), *Mikrogeophagus ramirezi* (3/31), and *H. bimaculatus* (2/31). Most affected fish originated from shopkeepers (15/31) and farmers (11/31). In 22 out of 31 fish, the fungal hyphae affecting skin and muscle were the primary finding. Two fish exhibited deeper lesions characterized by granulomatous coelomitis. Coinfections were observed with gastric granulomas suggestive of *Cryptobia* infection (5/31), digenetic trematodes (3/31), presumed *Epistylis* (2/31), intraluminal renal myxosporidians (1/31), and presumed *Ichthyophthirius multifiliis* (1/31) (Table S1).

#### 3.2.4. Viral Diseases

Twenty-three fish exhibited lesions consistent with *Lymphocystis Disease Virus* (LCDV) infection. These data are previously described and published in detail [14]. All cases occurred in *T. trichopterus* originating from farms, and no significant coinfections were identified (Table S1).

#### 3.2.5. Neoplastic Diseases

**Figure 4:**
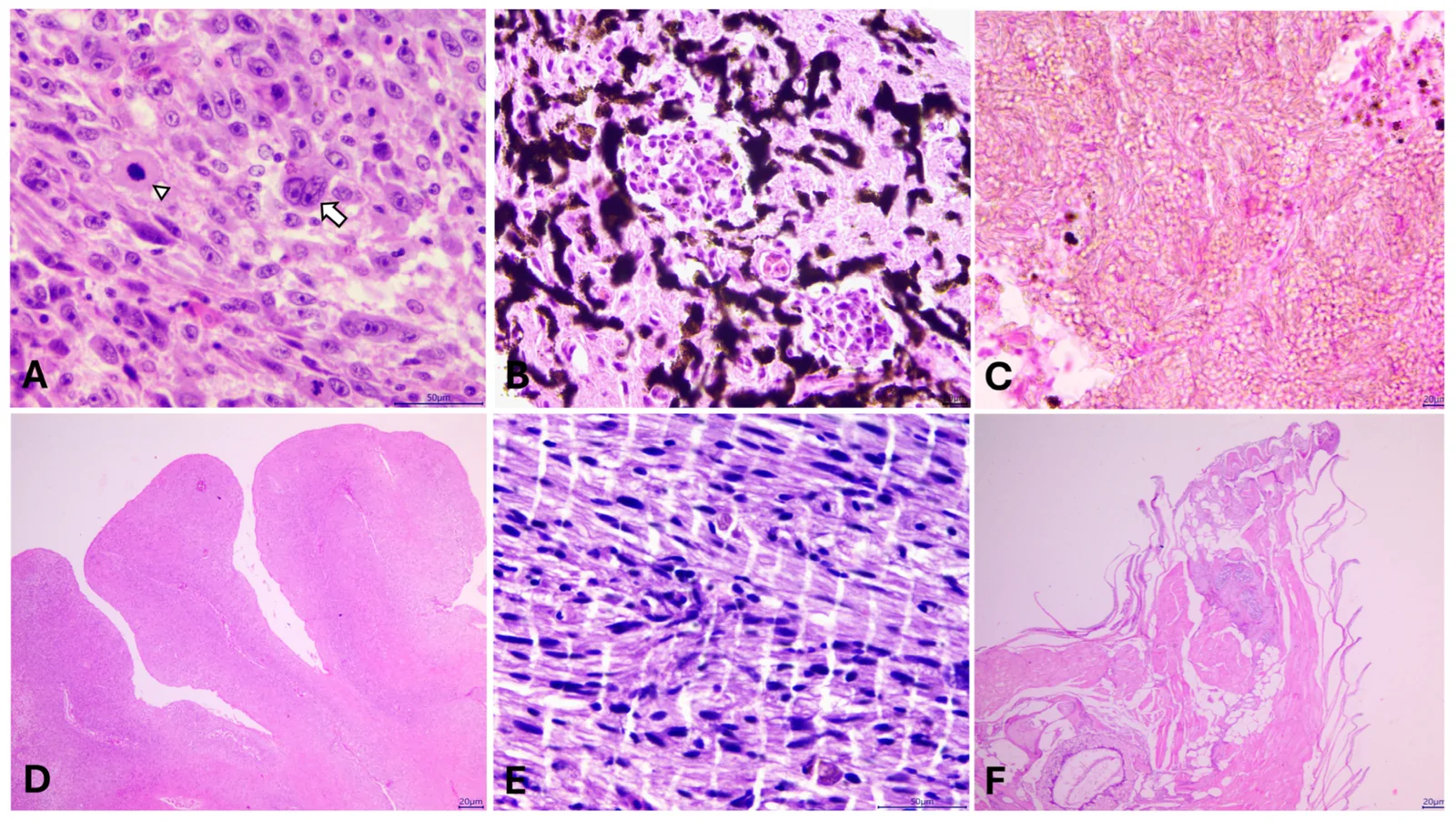
(**A**) Sarcoma in *Cyprinus carpio* composed of pleomorphic polygonal neoplastic cells, including binucleated (arrow) cells and atypical mitotic figures (arrowhead). H&E stain, 600× magnification. (**B**) Chromatophoroma in *B. splendens* composed of neoplastic chromatophores, including melanophores containing black intracytoplasmic pigment granules and cells with eosinophilic granular cytoplasm. H&E stain, 400× magnification. (**C**) Chromatophoroma in *B. splendens* composed predominantly of neoplastic iridophores containing abundant refractile brown cytoplasmic pigment. H&E stain, 600× magnification. (**D**) Papilloma in *C. carpio* exhibiting exophytic digitiform epithelial proliferations composed of neoplastic epidermal epithelial cells. H&E stain, 40× magnification. (**E**) Benign mesenchymal tumor in *C. auratus* composed of spindle-shaped cells arranged in interlacing bundles. H&E stain, 600× magnification. (**F**) Underdeveloped and tortuous caudal peduncle in *D. rerio* associated with malformation of the caudal lepidotrichia. H&E stain, 100× magnification.

Six cases were diagnosed as neoplasms. Among them, two were suggestive of sarcomas, two of chromatophoroma, one of benign mesenchymal neoplasia, and one of papilloma. The sarcoma cases occurred in *Cyprinus carpio* and *C. auratus* (Figure 4A), while the chromatophoroma cases were identified in *B. splendens* (Figure 4B,C). The papilloma was observed in *Cyprinus carpio* (Figure 4D), and the benign mesenchymal neoplasia in *C. auratus* (Figure 4E). These cases originated from different sources, and the tumors were located on various anatomical regions of the skin (Table 3).

#### 3.2.6. Congenital Diseases

Eight fish exhibited congenital malformations, all of which were *D. rerio* originating from reproduction in a laboratory facility (Table S1). The alterations consisted of malformations, deviations, and incomplete development of the caudal peduncle. Microscopically, the caudal peduncle and vertebral column showed marked disorganization and loss of muscle fibers, which were replaced by fibrous and adipose tissue (Figure 4F). Additionally, there was disorganization and loss of vertebral bone tissue and caudal lepidotrichia, resulting in atrophy and reduction in the caudal peduncle.

#### 3.2.7. Metabolic Diseases

Three cases presented exclusively metabolic alterations. One case of hepatic lipidosis was observed in *Balantiocheilos melanopterus* from a shopkeeper, characterized by marked vacuolization and occasional rupture of hepatocytes. In addition, two cases of granulomatous nephritis associated with nephrocalcinosis were diagnosed—one in *Paracheirodon axelrodi* from a shopkeeper and another in *X. maculatus* from a farm (Table S1).

#### 3.2.8. Coinfections

Lesions associated with concurrent parasitic infections, as well as complex cases presenting combined parasitic, bacterial, and fungal etiologies, were recorded (Table S1). Infections attributed to *Trichodina* spp. and digeneans, along with *Eustrongylides* sp. and *C. formosanus* were frequently identified in fish from farmers. In contrast, infestations by *Tetrahymena* sp. and *Camallanus* spp., together with cases suggestive of *Cryptobia* spp. and *Saprolegnia* spp., were primarily observed in shopkeeper-derived specimens (Table S1).

### 3.3. Multiple Correspondence Analysis

**Figure 5:**
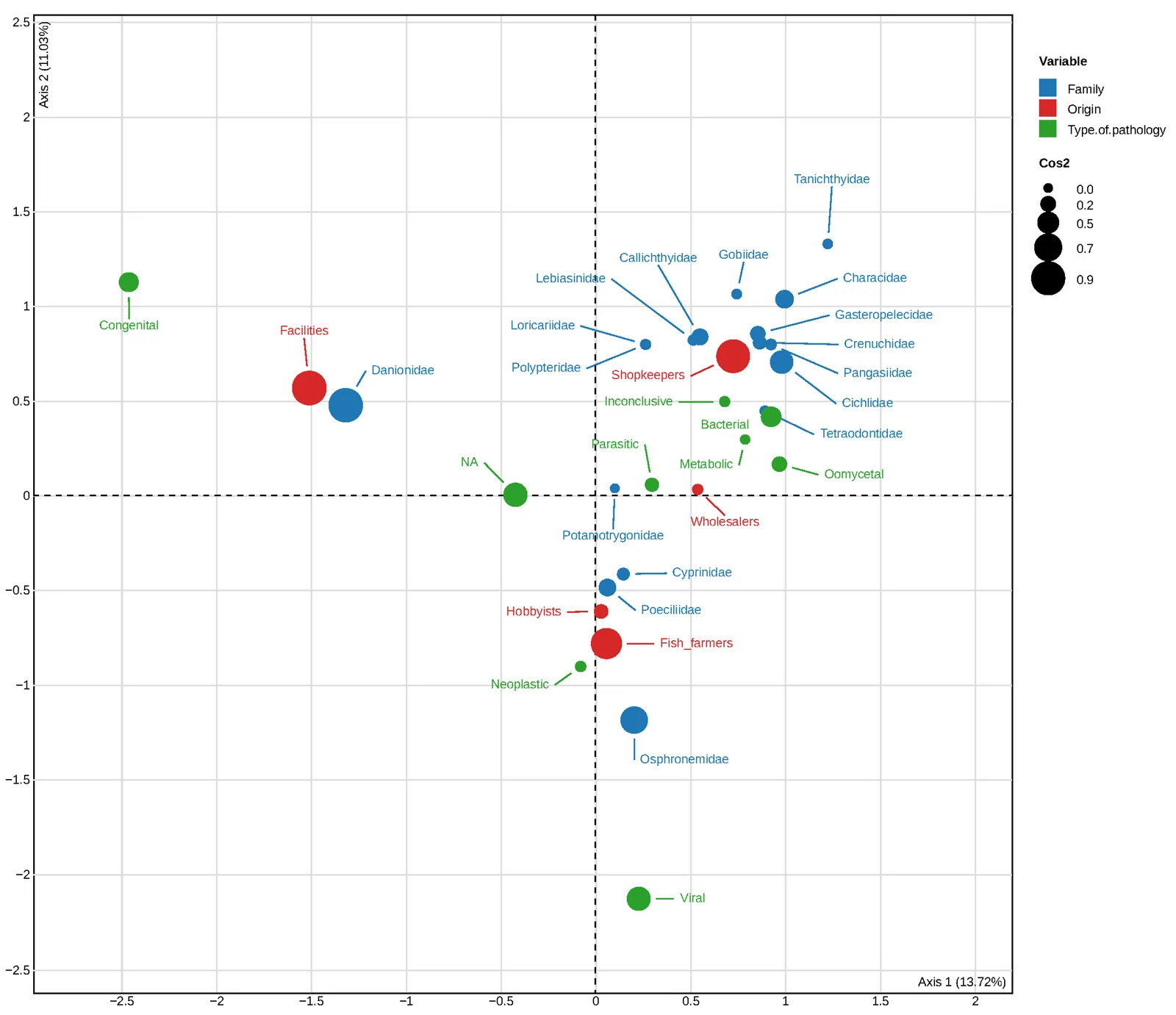
Multiple correspondence analysis of categorical variables fish family, sample origin, and type of disease detected. The variables were considered related when grouped together. The circle’s diameter represents the cos^2^ value (ranging from 0 to 1), demonstrating the variable’s level of association with the projected factor plane.

The multiple correspondence analysis revealed that fish originating from laboratory facilities belonged exclusively to the family Danionidae, which mostly showed no pathological alterations and occasionally parasitic diseases. Congenital malformations were identified solely in fish from laboratory facilities. The shopkeepers and wholesalers cases exhibited the greatest diversity of ornamental fish families, predominantly affected by bacterial, fungal, and parasitic diseases. Fish from farms and hobbyists mainly belonged to the families Poecilidae, Cyprinidae, and Osphronemidae. Among these origins, several parasitic diseases were identified, while neoplastic and viral diseases occurred exclusively in fish from these groups (Figure 5).

## 4. Discussion

There is a wide diversity of ornamental fish species cultured in Brazil, where current legislation allows the commercialization of at least 1226 species [1]. This scenario enabled the present study to evaluate a broad diversity of ornamental fish species (68 species), like other health surveillance studies on fish conducted in the country [6,7,25].

Histopathological findings were identified in approximately 50% of the samples evaluated. When compared with another histopathological survey of ornamental fish conducted in Australia, it was observed that the authors reported relatively higher rates, with 71.3% (77/108) of the fish showing histopathological alterations [19]. It should be considered that, in the present study, samples also included fish from laboratory facilities and farms where animals without clinical signs were submitted to the diagnostic service to assess their sanitary status, which may have contributed to the number of samples without detectable lesions. Regarding the etiological groups, parasitic and bacterial agents were predominant. These results are consistent with findings from other studies that employed histopathology as the main diagnostic approach for ornamental fish, in which parasitic diseases were the most frequent, followed by lesions associated with bacteria such as *Mycobacterium* spp., *Epitheliocystis*, and *Flavobacterium* spp. [19]. Similarly, a study on freshwater ornamental fish in Iran also identified parasitic infections as the most frequent pathological condition [26]. As histopathology was the diagnostic method employed and no microbiological or molecular techniques were used to further investigate the involvement of other pathogens, this may have contributed to the higher frequency of identification of parasitic etiologies. Studies conducted in Brazil have reported a high frequency of pathogens similar to those identified in the present survey [6,7,25]. Investigations on the parasitic fauna and histopathological alterations in freshwater ornamental fish farms in southern Brazil identified monogeneans, *Ichthyophthirius multifiliis*, trichodinids, digenetic trematodes, nematodes, cestodes, and branchiurans as the main agents [6]. A study focusing on the parasitological diagnosis of ornamental fish farms from different Brazilian states reported the predominance of monogenean trematodes, metacercariae, cestodes, nematodes, *Lernaea cyprinacea*, *Trichodinids*, *Piscinoodinium pillulare*, *Ichthyophthirius multifiliis*, diplomonad flagellates, *Ichthyobodo* sp., *Chilodonella* sp., and *Tetrahymena* sp. [7]. Similarly, various protozoan, monogenean trematode, arthropod, and nematode species have been identified as important parasites of cultured freshwater ornamental fish in Iran [27].

Digeneans are heteroxenous parasites that typically require mollusks and small fish as intermediate hosts before reaching their definitive hosts, which may include birds, mammals, or piscivorous fish. The most common digenetic trematodes in freshwater fish belong to the families Clinostomidae, Diplostomidae, Isoparorchiidae, Strigeidae, and Heterophyidae [28]. In Brazil, the importance of digenetic trematodes in ornamental fish has been well documented [6,7]. In the present study, numerous metacercariae were detected; however, histopathological morphology did not allow identification to the genus or species level. These cysts were mainly located in skeletal muscle, gills, liver, and coelomic cavity. Species-level identification of these agents by histopathology alone can be challenging and often require complementary parasitological analyses. In most cases, there was no associated inflammation, as the cyst capsule remained intact. This finding is consistent with the literature, which indicates that metacercariae generally do not compromise fish health; however, depending on their location, abundance, and the extent of morphological alterations caused by the parasite, they may be detrimental to the host [28]. In the present survey, the main ornamental fish species harboring unidentified digeneans included *C. strigata*, *Corydoras* spp., and *C. asellus*. These species are commonly sourced from wild-capture fisheries in rivers (Amazon basin) where environmental conditions favor the life cycle of digenetic trematodes [28,29]. In some cases, more precise identification was possible, including infections caused by *Centrocestus formosanus* and *Clinostomum* spp.

Lesions presumably caused by *Centrocestus formosanus* metacercariae were detected in the gills of different fish species. The affected gills exhibited lamellar thickening, nodule formation, and the development of a cartilaginous capsule surrounding the parasite, which represents a distinctive histopathological feature for the identification of this trematode [30,31]. *C. formosanus* has been reported in ornamental fish species from several countries, including Sri Lanka [32], Mexico [33], Iran [34], Thailand [35], and Brazil [36]. In Brazil, the known intermediate host of *C. formosanus* is the gastropod *Melanoides tuberculata* [37]. The parasite has been reported in various fish species such as *P. reticulata* [38], *Australoheros facetus* [39], and *Xiphophorus* spp. [36,40] across multiple regions of the country. In addition to morphological parasitological identification, molecular analyses have also been employed to confirm the presence of this trematode [36,40]. In the present study, *D. rerio* and *C. auratus*, both exotic species, were apparently the main hosts affected by *C. formosanus*. Most of these cases originated from farms, reflecting low-technology farming conditions where fish are maintained in earthen ponds with potential exposure to *M. tuberculata* and various bird species that act as definitive hosts, facilitating the completion of the parasite’s life cycle [31].

Another important digenetic trematode was presumably identified in the present study as *Clinostomum* spp. Similarly to other digeneans, *Clinostomum* spp. requires snails as intermediate hosts and piscivorous birds as definitive hosts [41,42]. During the metacercarial stage, these trematodes can cause tissue damage and impair the reproductive performance of infected fish [43]. In Brazil, the parasite has been reported in piscivorous birds and in 31 freshwater fish species [44]. In the present study, presumed *Clinostomum* spp. infection was diagnosed in *P. scalare* and *Poecilia reticulata*, species in which parasitological studies have previously confirmed infection in different regions of the country [41,45]. The lesions were observed mainly in the skin and skeletal musculature, although the parasite can also occur in other anatomical sites, such as the gills, mesentery, and intestine [43]. The affected fish originated from farms and wholesalers, suggesting that these animals were exposed to environmental conditions favorable to the completion of the parasite’s life cycle, either during production or in their natural habitats.

Monogenean were also identified in the present survey. This group of parasites is among the most frequently reported in Brazilian ornamental aquaculture and infects a wide range of host species [6,7,46,47]. In the present study, *C. auratus* was the most affected species, with most infected individuals originating from farms. This finding is consistent with previous national studies, which indicate that low-technology farming systems favor the dissemination of these parasites [7,48]. As monogenean infestations are well-known among ornamental fish producers, distributors and retailers often implement control measures, including the use of salt baths. We suggest that such management practices may have contributed to the relatively lower frequency of monogeneans observed in this study compared with reports from other regions of the country [7,25].

In the present study, capillariid nematodes were detected in *P. reticulata* and *Danio rerio*. Several species of capillariids are known to infest ornamental fish such as *P. reticulata*, *P. scalare*, *Danio rerio*, and *Puntigrus tetrazona* [49]. Among the most important capillariids, *Pseudocapillaria tomentosa* stands out as a pathogenic nematode that infects *D. rerio*, representing a significant health challenge in research facilities [49,50,51]. In the examined samples, the capillariids were found either embedded in the intestinal mucosa or free within the intestinal lumen; however, no marked inflammatory or proliferative changes were observed. The literature reports that this parasite is commonly located in the rectum and posterior intestinal segments [52], where it can be associated with inflammatory, proliferative, or even neoplastic lesions [50]. In the present survey, most fish harboring capillariids originated from research facilities and farms, which aligns with previous data indicating *P. tomentosa* infestation in approximately 15–20% of *D. rerio* research facilities [53,54]. Another relevant species is *Capillaria pterophylli*, a nematode that parasitizes the intestines of cichlids, particularly *P. scalare*, and has been reported in Brazil [55]. Specific parasitological analyses are required to differentiate between these capillariid species.

*Camallanus* spp. was an important nematode identified in *P. reticulata* in the present study with support of the parasitological morphology identification. Species of *Camallanus* can infect the gastrointestinal tract of cichlids, poeciliids, and other ornamental fish species. Copepods or other crustaceans serve as intermediate hosts in the parasite’s life cycle [56]. In Brazil, *C. maculatus* has been reported in *X. maculatus* [57], and *B. splendens* [58], while *C. tridentatus* was reported in *Arapaima gigas* [59]. In the present study, the nematodes were found within the intestinal lumen, free in the coelomic cavity, or near the anus, which is consistent with the lesion sites reported in experimental studies [58]. Most cases compatible with *Camallanus* spp. infection originated from shopkeepers, highlighting the challenges in controlling this parasite among traded ornamental fish. Similar findings have been reported in previous studies emphasizing the significance of *Camallanus* spp. in ornamental aquaculture [60,61,62].

In the present study, *Eustrongylides* spp. were diagnosed in *D. rerio*, with support from parasitological morphological identification. However, these nematodes can infect a wide range of fish species, including ornamental fish such as *P. reticulata*, *D. rerio*, and *P. scalare* [56]. The three main species are recognized within this genus were *E. excisus*, *E. tubifex*, and *E. ignotus*, the latter two having already been reported in Brazil in both birds and fish [63]. In the country, *Eustrongylides* spp. has been described in *Hoplias malabaricus* [64,65], *Polypterus endlicheri* [66], and *Triportheus angulatus* [67]. Outbreaks have also been reported in *D. rerio* acquired from pet shops in Brazil [68,69]. In the present survey, nematode larvae were found within the coelomic cavity, compressing adjacent organs and impairing the swimming ability of affected fish. These findings are consistent with previous outbreak reports in the country [68,69]. The cases identified in this study originated from farms and research facilities, differing from other reports in which *D. rerio* were purchased from pet shops to establish research colonies, indicating the broad distribution of this parasite in Brazil [68,69]. It is noteworthy that *Eustrongylides* spp. has a complex life cycle, with fish-eating birds as definitive hosts, and may infect accidental hosts such as amphibians, reptiles, and mammals. This includes a potential zoonotic risk to humans through the consumption of raw fish harboring infective larvae [70].

The protozoan infections indicated by the microscopic examination in the present survey are among the most prevalent pathogens reported in other studies on the health of ornamental fish in Brazil [6,7,46,47]. *Tetrahymena* spp. is a pyriform ciliate protozoan, with *T. corlissi* recognized as a highly pathogenic species that causes severe disease in *P. reticulata*, although it can also infect other important ornamental fish species [71]. *Tetrahymena pyriformis* has also been reported to cause mortality in *Puntius tetrazona* [72]. In the present study, *P. reticulata* was the main species affected by *Tetrahymena* spp. The necrotizing skin and muscle lesions, which produce the popularly known “white spot” appearance observed in affected fish, were consistent with descriptions from previous studies on the pathogenicity of this protozoan [73]. The microscopic appearance, tissue distribution, and systemic manifestation of the parasites observed in the present study are consistent with descriptions in previous reports [71,73]. The infected fish primarily originated from shopkeepers and farms, which aligns with studies highlighting the prevalence of *Tetrahymena* spp. infections in *P. reticulata* from both shopkeepers [25] and farms [7]. The difficulty in controlling and preventing this protozoan in *P. reticulata* and other ornamental species contributes to its persistence in culture environments.

*Epistylis* is a sessile, ciliated, and ectocommensal protozoan [74]. In the present study, the suspected cases of this parasite infection were most frequently detected in *Cyprinus carpio* and coinfection with oomycete and bacterial agents, consistent with previous reports [74,75]. It has been suggested that *Epistylis* is not primarily pathogenic and that bacteria and fungi represent the primary agents initiating lesions at the peduncular insertion site, a pattern also noted in the histological sections examined [74]. The fact that most infected fish originated from farms reflects the favorable conditions for the maintenance of the protozoan in farm environments, where fish are commonly raised in earthen ponds. Some studies have also highlighted the potential role of crustaceans as mechanical vectors of *Epistylis* spp. [75,76].

*Cryptobia iubilans* and other kinetoplastid protozoans are recognized causes of high mortality in *S. discus* and other cichlid species due to granulomatous gastritis [77,78]. Consistent with the literature, the species of fish showing lesions compatible with this agent in the present survey were *Aulonocara* spp. and *S. discus*. Reports in *S. discus* exist from the United States [77] and Trinidad and Tobago [78]. In this survey, classic gastric granulomas were the predominant findings, although intestinal and hepatic granulomas were also observed [77,78].

*Ichthyophonus* spp. is a mesomycetozoean organism exhibiting both fungal and metazoan characteristics [79]. The principal pathogenic species is *Ichthyophonus hoferi*, known to cause a systemic granulomatous disease [80]. In the present study, suspected cases occurred mainly in *P. weitzmani* and *D. rerio*. No records of *Ichthyophonus* spp. have been reported in Brazil to date. In most cases in our survey, the agent was detected histopathologically without associated inflammation, with only occasional histiocytic or granulomatous infiltrates in ruptured cysts, consistent with previous descriptions [80].

*Pleistophora hyphessobryconis* is a microsporidian and an obligate intracellular parasite. It is known as the causative agent of “tetra disease,” first identified during an outbreak in *Paracheirodon innesi* [81]. It is now recognized that *P. hyphessobryconis* can infect species from the families Cyprinidae, Cyprinodontidae, Poecilidae, and Cichlidae, particularly those originating from the Amazon River basin [82]. Although *P. hyphessobryconis* is associated with Amazonian fish, the only report involving native Brazilian species refers to hybrid jundiara (*Leiarius marmoratus × Pseudoplatystoma reticulatum*) [82]. In the present survey, cases suggestive of *Pleistophora* sp. infection occurred in *C. strigata*, members of the family Gasteropelecidae, which are of Amazonian origin and consistent with published literature. This is the first report of this agent in this species. Spore-induced lesions within muscle fibers, accompanied by myonecrosis and inflammation surrounding ruptured cysts, matched descriptions from previous outbreaks [82,83]. All infected fish originated from shopkeepers who ordered stock from northern Brazil for resale. These fish are collected from the wild and therefore remain exposed to a wide range of environmental pathogens, which explains why all of them were coinfected with metacercariae of digenetic trematodes.

In *C. strigata* obtained from shopkeepers, *C. asellus* from a wholesaler, and a *C. auratus* from a farm, intraluminal structures were detected within renal tubules that were suggestive of renal myxosporeans. Myxosporeans are multicellular cnidarian parasites and are among the most significant pathogenic agents in fish. They form spores and vegetative stages capable of inducing hypertrophy, xenoma formation, and degeneration of infected cells [84]. Families such as Ortholineidae include coelozoic *Ortholinea* spp. that infect the urinary tract of marine and freshwater fish, including *Ortholinea fluviatilis* and *Ortholinea saudii* [85,86]. Another family, Sphaerosporidae, includes species such as *Sphaerospora renicola*, which infects the kidney and swim bladder of *Cyprinus carpio*, and *Hoferellus carassi*, an important pathogen of the renal tubules in *C. auratus* [84]. Although these are the suspected agents in the cases of this study, molecular analyses are required to confirm the species identity of these agents.

Lesions compatible with or suggestive of mycobacteriosis were identified in multiple species and at all levels of the ornamental fish production chain. Mycobacteriosis is one of the most significant diseases of fish, representing the most common chronic bacterial infection and a major challenge for ornamental fish production [87]. The causative bacteria, known as nontuberculous mycobacteria (NTM), are ubiquitous in aquatic environments [9]. *M. marinum*, *M. chelonae*, *M. fortuitum*, *M. gordonae*, and *M. peregrinum* have been isolated from ornamental fish worldwide [88,89]. *M. chelonae*, *M. fortuitum*, *M. gordonae*, and *M. intracellulare* are considered potentially zoonotic and have been associated with skin lesions and occupational exposure to infected fish and environments [9,90,91]. The distribution of lesions in this study also aligns with the literature: the most frequently affected organs were the kidney (76.08%; 35/67), liver (35.82%; 24/67), spleen (25.37%; 17/67), and intestine (16.41%; 11/67). These frequencies are comparable to a study from the Czech Republic reporting the following distribution: kidney (81.2%), digestive tract (54.1%), liver (48.2%), and spleen (45.9%) [88]. Of the 56 samples subjected to Fite–Faraco staining, 24 (42.86%; 24/56) tested positive. This finding is consistent with other studies, such as other surveys reporting 51.1% positivity (96/188) [88]. In contrast, an analysis of 54 park pond fish in São Paulo detected only 8 fish positives for acid-fast bacteria (14.81%; 8/54) [92]. For detecting acid-fast bacteria (AFB) in fish granulomas, Fite–Faraco staining is preferable to the traditional Ziehl–Neelsen method [93]. The classical Ziehl–Neelsen technique often fails to stain bacilli intensely red because the low mycolic acid content in these organisms is removed by xylene during deparaffinization [8]. Immunohistochemical and molecular assays can be applied to investigate granulomas and confirm mycobacteriosis [8,94,95].

Columnaris disease is an important cause of mortality in wild, farmed, and ornamental fish. The genus *Flavobacterium* includes several significant species, such as *F. columnare*, *F. covae*, *F. davisii*, and *F. oreochromis* [96]. In Brazil, a recent study on farmed fish reported the presence of *F. davisii*, *F. covae*, and *F. oreochromis* in the samples analyzed [97]. No studies were found identifying the most frequent *Flavobacterium* spp. in ornamental fish under this revised classification. The pathogenesis of this bacterium remains incompletely understood, but evidence suggests that its thick capsule and the presence of chondroitin AC in its cell wall enable destruction of chondroitin sulfate and hyaluronic acid in host connective tissues. These features may contribute to the bacterium’s ability to respond to chemotactic signals, adhere to host surfaces, and form aggregates [98]. The action of these enzymes may explain the extensive areas of necrosis with minimal to no associated inflammatory infiltrate observed in the fish examined in the present survey.

Thirty-one fish exhibited lesions suggestive of oomycete infection. The importance of oomycetes in the national ornamental aquaculture sector is well recognized, with lesions attributed to members of the order *Saprolegniales*, particularly species of the genera *Saprolegnia* and *Achyla*, being considered the most relevant [5]. These organisms are saprophytic oomycetes that typically act as opportunistic pathogens in preexisting lesions. Fish in the present study showed macroscopic ulcerative dermatitis with accumulations of whitish, cotton-like, exophytic material, a gross presentation characteristic of oomycete hyphae [5,99]. In addition to this pattern, large solitary skin ulcers were observed in *Trichogaster lalius*. Microscopically, hyphae were associated with necrosis and histiocytic or granulomatous inflammatory infiltrates, consistent with previous reports [100,101]. Although the most likely etiological agents are *Saprolegnia* spp., the involvement of *Achyla* spp. cannot be excluded. Molecular analyses for the identification of oomycetes directly from tissue samples remain challenging, with pathogen isolation followed by DNA extraction being the most employed approach [102]. Therefore, the development and expansion of molecular techniques for direct extraction and identification from tissue samples are of considerable importance [100].

In Brazil, several viruses associated with fish diseases have been reported, including spring viremia of carp virus [103], Lymphocystis virus [11,12,13], carp edema virus [15], Cyprinid herpesvirus 1 [16], and infectious spleen and kidney necrosis virus [104]. However, histopathological, ultrastructural, and molecular investigation of the animals collected in this survey enabled the first report of Lymphocystis virus in *T. trichopterus* in the country [14].

Various neoplasms have been identified in ornamental fish [105]. Physical, chemical, environmental, and biological factors may contribute to tumor development in these species. Chemical pollution and ultraviolet radiation are frequently cited as potential risk factors for neoplasia [106]. Biological factors may also play a role, including infection with *Cyprinid herpesvirus* 1 in the development of papillomas [107] and cutaneous squamous cell carcinoma in *Cyprinus carpio* [108], retrovirus-associated proliferative diseases [109], and infestation with *Pseudocapillaria tomentosa* in gastrointestinal neoplasms of zebrafish [50]. Sarcoma, a malignant tumor of mesenchymal origin, has been considered the most prevalent tumor type (85%) in retrospective studies of neoplastic diseases in fish [110], although other studies report epithelial and neuroectodermal tumors as more frequent [111]. Despite this variability, several investigations agree that the most common tumors originate from the skin [109,110,111]. Commonly affected sites include multiple body regions, the eye and periorbital fascia, head, and neck [110]. Species with the highest tumor frequency include *Cyprinus rubrofuscus* and *C. auratus* [110]. Chromatophoromas are neuroectodermal neoplasms arising from chromatophores. A recent study described the histological and ultrastructural features of iridophoromas in *B. splendens* in Brazil, reporting higher frequency in fish older than six months, with lesions most commonly affecting the trunk and tail or occurring at multiple sites [18]. Papillomas are benign epithelial neoplasms reported in several species [112,113]. *Cyprinid herpesvirus* 1 is associated with proliferative lesions in *C. carpio*, including papilloma formation [107]. Temperatures below 20 °C favor the development of ectodermal plaques (epithelial hypertrophy and hyperplasia) in carp infected with CyHV-1 [114], whereas higher temperatures are expected to induce lesion regression [115,116].

Malformations in fish have a multifactorial etiology and may be congenital or acquired. Nutritional imbalances, chemical compounds, abrupt temperature shifts, altered photoperiods, genetic mutations, high levels of inbreeding or consanguinity, predator attacks, infections, and parasitic infestations may contribute to skeletal or soft-tissue deformities [117]. In the present survey, congenital malformations were diagnosed in eight *D. rerio* produced in a facility, and these animals lacked any parasitic or inflammatory lesions. The incubation and hatching processes of *D. rerio* eggs are highly sensitive; in principle, the eggs were maintained within the optimal temperature range for development (26–28.5 °C). Previous studies reported that tail malformations arise at 32.5 °C within the first 24 h post-fertilization, whereas temperatures between 34.5 and 36.5 °C lead to more severe alterations, including cranial and cardiovascular defects [118]. Thus, deviations in hatching temperature may be associated with the malformations identified in the present sample. 

Two cases of severe nephrocalcinosis were identified in fish. Nephrocalcinosis is a condition with a poorly defined and multifactorial etiology [119]. Several factors have been associated with this condition, including elevated carbon dioxide concentrations in the water, dry feeds (particularly in marine species), and inadequate calcium or magnesium levels in the diet. Although nephrocalcinosis may occur in free-living animals, the most severe cases have been reported in fish under intensive culture conditions. Exposure to high carbon dioxide levels is frequently linked to metabolic acidosis and subsequent calcium precipitation within the urine [119,120].

Anatomopathological examination is a highly valuable tool for the diagnosis of diseases in ornamental fish. Histopathology is a relatively inexpensive technique that allows the integration of clinical, epidemiological, microbiological, and molecular findings with the morphofunctional alterations observed in affected animals. Although considered a traditional diagnostic method, histopathology remains indispensable for establishing causal relationships between diseases and their etiological agents, as well as for elucidating disease pathogenesis [121]. Nevertheless, it should be emphasized that different pathogens may induce similar lesions, and morphological features alone often do not permit species-level identification. Consequently, diagnostic errors or bias may occur when histopathological findings are interpreted in isolation. Therefore, molecular and microbiological analyses should be incorporated whenever possible to improve the accuracy of etiological diagnoses.

Our analytical methodology also had limitations in identifying co-infections, as interpreting the significance of concurrent processes can be influenced by sample availability and representativeness and can also be subjective, relying on the pathologist’s conclusions. It is also important to note that subtle disease-associated lesions (for example, brain lesions caused by bacterial or viral infections), or diseases caused by unknown or poorly described pathogens (such as viral diseases) could influence the diagnostic sensitivity and the frequency of the diseases in a cohort similar to the one evaluated in this study. Unfortunately, these challenges are inherent realities for diagnostic services, even those equipped with all diagnostic tools.

When the distribution of diseases was evaluated according to origin, parasitic diseases were found to predominate across all fish sources. Among farms, the main parasites were presumed *C. formosanus* and *Epystilis* spp.; among shopkeepers, digenetic trematodes (species not identified) and presumed *Tetrahymena* spp. were most common. In facilities, capillariids were the primary parasites, while among aquarists, presumed *Piscinoodinium pillulare* was prevalent. In wholesalers, presumed *Ichthyophthirius multifiliis* infection was most frequent. The occurrence of these pathogens is influenced by multiple risk factors, including the presence of specific vectors (as in cases of *C. formosanus* infection, for example), stress and transportation (particularly for diseases in shopkeepers and wholesalers), and suboptimal water conditions, stress, and persistent bacterial infections (notably among hobbyists). Notably, the greatest diversity of parasitic agents and the highest number of fish with lesions suggestive of bacterial diseases were observed among shopkeepers. This may be attributed to the fact that shopkeepers gather fish from a wider variety of species and regions, and these fish are more likely to exhibit clinical signs after experiencing the stress of transportation and acclimatization.

## 5. Conclusions

Ornamental fish are exposed to a wide range of pathological conditions, with parasitic alterations being the most frequent. Histopathology is an essential tool for detecting and characterizing pathogens. This approach enabled the possible identification of agents previously unreported in the country, such as presumed *Ichthyophonus* spp. and renal myxosporeans. In addition, pathogens were documented in host species not previously reported in Brazil, including *Pleistophora* sp. in *C. strigata.* However, molecular analyses should be performed to confirm the etiological diagnoses. Parasitic alterations predominated across all fish sources; nonetheless, bacterial, oomycete, viral, neoplastic, congenital, and metabolic diseases also occur in the diagnostic routine of ornamental fish. The present study is expected to support the identification of pathogens and associated pathological findings, thereby encouraging further research employing histopathology to improve ornamental fish health in Brazil. Best management practices, quarantine protocols, and targeted therapeutic and antiparasitic treatments are strategies that can be implemented to mitigate the infectious and parasitic agents identified in this study within commercial ornamental fish aquaculture and throughout its entire production chain.

## Figures and Tables

**Table 1:** Total number of exotic and native ornamental fish organized by order, family, genus, or species, received between 2019 and 2023 for pathological examination.

Order (*n*)	Family (*n*)	Genus/ Species (*n*) Exotic (E)/Native (N)
**Cypriniformes (206)**	Danionidae (143)	*Danio rerio* (135) (E)
		*Danio margaritatus* (6) (E)
		*Danio erythromicron* (1) (E)
		*Trigonostigma heteromorfa* (1) (E)
	Cyprinidae (62)	*Carassius auratus* (34) (E)
		*Cyprinus carpio* (21) (E)
		*Oliotius oligolepis* (2) (E)
		*Puntigrus tetrazona* (2) (E)
		*Puntius titteya* (2) (E)
		*Balantiocheilos melanopterus* (1) (E)
	Tanichthyidae (1)	*Tanichthys albonubes* (1) (E)
**Cyprinodontiformes (150)**	Poeciliidae (150)	*Poecilia reticulata* (73) (E)
		*Xiphophorus hellerii* (28) (E)
		*Poecilia sphenops* (27) (E)
		*Xiphophorus maculatus* (22) (E)
**Anabantiformes (97)**	Osphronemidae (97)	*Trichogaster trichopterus* (33) (E)
		*Trichogaster lalius* (32) (E)
		*Betta splendens* (30) (E)
		*Ctenopoma acutirostre* (1) (E)
		*Trichogaster* spp. (1) (E)
**Cichliformes (63)**	Cichlidae (63)	*Pterophyllum scalare* (23) (N)
		*Mikrogeophagus ramirezi* (8) (E)
		*Symphysodon discus* (7) (N)
		*Aulonocara* spp. (6) (E)
		*Chindongo demasoni* (3) (E)
		*Aulonocara maylandi* (2) (E)
		*Aulonocara nyassae* (2) (E)
		*Maylandia estherae* (2) (E)
		*Rubricatochromis bimaculatus* (2) (E)
		*Aulonocara baenschi* (1) (E)
		*Dimidiochromis compressiceps* (1) (E)
		*Laetacara curviceps* (1) (N)
		*Melanochromis auratus* (1) (E)
		*Otopharynx lithobates* (1) (E)
		*Placidochromis phenochilus* (1) (E)
		*Protomelas fenestratus* (1) (E)
		*Rocio octofasciata* (1) (E)
**Characiformes (61)**	Acestrorhamphidae (21)	*Megalamphodus eques* (9) (N)
		*Paracheirodon axelrodi* (4) (N)
		*Paracheirodon simulans* (4) (N)
		*Hyphessobrycon amandae* (1) (N)
		*Megalamphodus erythrostigma* (1) (N)
		*Petitella rhodostoma* (1) (N)
		*Thayeria boehlkei* (1) (N)
	Gasteropelecidae (19)	*Carnegiella strigata* (19) (N)
	Crenuchidae (13)	*Crenuchus spilurus* (6) (N)
		*Poecilocharax weitzmani* (6) (N)
		*Characidium fasciatum* (1) (N)
	Lebiasinidae (6)	*Nannostomus marginatus* (5) (N)
		*Copella arnoldi* (1) (N)
	Characidae (2)	*Aphyocharax anisitsi* (2) (N)
**Siluriformes (48)**	Callichthyidae (36)	*Aspidoras pauciradiatus* (7) (N)
		*Corydoras spp.* (7) (N)
		*Corydoras panda* (4) (N)
		*Corydoras elegans* (3) (N)
		*Corydoras hastatus* (3) (N)
		*Corydoras serratus* (3) (N)
		*Aspidora spp.* (2) (N)
		*Corydoras duplicareus* (2) (N)
		*Corydoras pygmaeus* (2) (N)
		*Corydoras julii* (2) (N)
		*Corydoras adolfoi* (1) (N)
	Loricariidae (7)	*Baryancistrus xanthellus* (3) (N)
		*Otocinclus affinis* (3) (N)
		*Baryancistrus spp* (1) (N)
	Pangasiidae (5)	*Pangasianodon hypophthalmus* (5) (E)
**Tetraodontiformes (5)**	Tetraodontidae (5)	*Carinotetraodon travancoricus* (2) (E)
		*Colomesus asellus* (3) (N)
**Gobiiformes (2)**	Gobiidae (2)	*Elacatinus oceanops* (1) (E)
		*Brachygobius doriae* (1) (E)
**Myliobatiformes (2)**	Potamotrygonidae (2)	*Potamotrygon motoro* (2) (E)
**Polypteriformes (1)**	Polypteridae (1)	*Polypterus senegalus* (1) (E)
**TOTAL**		635

**Table 2:** Diseases/pathogens diagnosed by histopathology in ornamental fish from 2019 to 2023.

Disease Category	Disease/Pathogen	N	Frequency	
	(Primary Diseases + Coinfections *)		Within Category (%)	Among Total Cases (%)
Parasitic	Unidentified digenean	34	15.45%	5.35%
	Presumed *Centrocestus formosanus*	32	14.55%	5.04%
	Monogeneans	29	13.18%	4.57%
	Presumed *Tetrahymena* spp.	18	8.18%	2.68%
	Presumed *Epistylis* spp.	14	6.36%	2.20%
	Presumed *Cryptobia* spp.	13	5.90%	2.05%
	*Camallanus* spp.	12	5.45%	1.89%
	Capillariids	12	5.45%	1.89%
	Presumed *Clinostomum* spp.	11	5%	1.73%
	Presumed *Trichodina* spp.	9	4.09%	1.42%
	*Eustrongylides* spp.	8	3.64%	1.26%
	Presumed *Ichthyophonus* spp.	8	3.64%	1.26%
	Presumed *Piscinoodinium pillulare*	8	3.64%	1.26%
	Presumed *Ichthyophthirius multifiliis*	4	1.82%	0.63%
	Presumed *Pleistophora* sp.	3	1.36%	0.47%
	Suggestive of renal myxosporidians	3	1.36%	0.47%
	Suggestive of amoebae	1	0.45%	0.16%
	Presumed *Henneguya* spp.	1	0.45%	0.16%
	Presumed *Calyptospora* spp.	1	0.45%	0.16%
Total (Parasitic)		220	34.65%	
Bacterial	Compatible with or presumably mycobacteriosis	67	87.01%	10.55%
	Presumed columnaris disease	10	12.99%	1.57%
Total (bacterial)		77	12.13%	
Fungal/Oomycetal	Presumed water mold infections	31	4.88%	
Viral	*Lymphocystis Disease Virus*	23	3.62%	
Congenital	Caudal peduncle malformation	8	1.26%	
Neoplastic	Chromatophoroma	2	33.33%	0.31%
	Sarcoma	2	33.33%	0.31%
	Papilloma	1	16.67%	0.16%
	Benign mesenchymal tumor	1	16.67%	0.16%
Total (neoplastic)		6	0.94%	
Metabolic	Lipidosis	1	33.33%	0.16%
	Nephrocalcinosis	2	66.66%	0.31%
Total (metabolic)		3	0.47%	

* The column “Disease/Pathogen” includes the sum of fish in which the respective agent represented either the main lesion or occurred as part of a coinfection. The “Disease category” frequency was calculated by dividing the number of fish affected by that specific disease/pathogen by the total number of fish presenting diseases within the same category. The “Total frequency” corresponds to the number of fish affected by each agent/disease divided by the total number of fish analyzed in the study (*n* = 635).

**Table 3:** Neoplastic diseases in ornamental fish in survey study.

Species	Source	Morphological Diagnosis	Location	Observations
*Cyprinus carpio*	Hobbyist	Sarcoma	Dorsum (skin)	Biopsy and necropsy. Markedly infiltrative and non-metastatic tumor
*Carassius auratus*	Farm	Sarcoma	Lateral trunk (skin)	Histologically well-differentiated but locally infiltrative tumor.
*Betta splendens*	Shopkeeper	Chromatophoroma	Head (skin)	Along with granulomas associated with Ziehl–Neelsen-positive bacilli. Mycobacteriosis.
*Betta splendens*	Hobbyist	Chromatophoroma	Dorsum (skin)	Metastasis to the testes.
*Cyprinus carpio*	Hobbyist	Papilloma	Mouth (skin)	Biopsy.
*Carassius auratus*	Farm	Benign mesenchymal tumor	Lateral trunk (skin)	Histologically well-differentiated, encapsulated, and expansive tumor.

## Data Availability

The original contributions presented in this study are included in the article/Supplementary Material. Further inquiries can be directed to the corresponding author.

## Supplementary Materials

The following supporting information can be downloaded at: https://www.mdpi.com/article/10.3390/ani16203164/s1, Table S1: Diseases diagnosed in ornamental fish from 2019 to 2023, organized according to the source of the fish. Table S2. Detailed summary of parasitic diseases diagnosed in the survey, indicating the main affected fish species and their respective sources.

## Abbreviations

The following abbreviations are used in this manuscript:

UFMG	Universidade Federal de Minas Gerais
EV-UFMG	Veterinary School at the Federal University of Minas Gerais (EV-UFMG)
CEUA	Animal Ethics Committee
SISGEN	National System for the Management of Genetic Heritage and Associated Traditional Knowledge
MULTILAB	Laboratory of the Multidisciplinary Animal Research Unit
ICB	Institute of Biological Sciences
MCA	Multiple correspondence analysis
LCDV	*Lymphocystis disease* virus
