# Supplementary material for: Histopathological Assessment of Diseases in Ornamental Fish from 2019 to 2023 in Minas Gerais State, Brazil
Source: Animals (Basel). 2026 Oct 9;16(20):3164. doi: 10.3390/ani16203164 (PMC13700644; doi:10.3390/ani16203164)
Supplement: Supplementary file 1 [file animals-16-03164-s001.zip › Supplementary Table S1_v2.pdf]

Supplementary Table S1. Diseases diagnosed in ornamental fish from 2019 to 2023, organized according to the source of the fish

Source (n)	Type of disease (n)	Etiological agent (n)	Coinfection/ Details (n)
**Farmer (235)**	Parasitary (72)	Presumed *Centrocestus formosanus* (13)	*C. formosanus* + Unidentified digeneans (1)
		Presumed *Epystilis* spp. (12)	*Epystilis* spp. + *Saprolegnia* (3)<br>*Epystilis* spp + metacercariae of *C. formosanus* (2)
		Presumed *Clinostomum spp.* (8)	
		Presumed *Trichodina* spp. (8)	*Trichodina* spp. + digeneans (8)
		Eustrongylides spp. (7)	*Eustrongylides* + *C. formosanus* (6)<br>*Eustrongylides* + C. *formosanus* + compatible with flavobacteriosis (1)
		Monogeneans (7)	Unidentified monogeneans + *C. formosanus* (2)<br>Unidentified monogeneans + suggestive of renal myxosporean (1)
		Presumed *Tetrahymena* spp. (7)	
		Capillariids (4)	
		Presumed *Cryptobia* spp. (4)	*Cryptobia* spp. + unidentified monogeneans (3)
		Presumed *Piscinoodinium pillulare (1)*	
		Unidentified digeneans (1)	
	Viral (23)	*Lymphocystis disease* virus (23)	
	Bacterial (13)	Compatible with or presumably mycobacteriosis (10)	*Mycobaterium spp. (Fite Faraco positive) (2)*<br>Compatible with of mycobacteriosis + unidentified monogeneans (2)
		Compatible with flavobacteriosis (3)	Compatible with flavobacteriosis + *C. formosanus* (1)
	Oomycete (11)	Presumed Water mold disease (11)	Water mold disease + compatible with flavobacteriosis (1)
	Neoplastic (2)	Sarcoma (1)	Sarcoma + *C. formosanus* (1)
		Benign mesenchymal tumor (1)	Benign mesenchymal tumor + granulomas + *Trichodina* spp. + unidentified monogeneans (1)
	Metabolic (1)	Nephrocalcinosis with granulomatous nephritis (1)	
	Inconclusive (3)	Severe necrotizing and lymphohistioplasmacytic myositis (1)	
		Focal granuloma (1)	
		Hepatic megalocytosis (1)	
	No lesions (110)		
**Shopkeeper (206)**	Parasitary (75)	Unidentified digeneans (24)	Unidentified digenean + Capillariids (1)
		Presumed *Tetrahymena* spp. (10)	*Tetrahyena* + compatible with *Camallanus* spp. (5)
		Presumed *Cryptobia* sp. (9)	Suggestive of *Criptobia* spp. + ulcerative dermatitis (1)<br>Suggestive of *Criptobia* spp + *Saprolegnia* spp. (5)
		Presumed *Ichthyophonus* spp. (6)	Compatible of mycobacteriosis (3)
		Unidentified monogeneans (6)	

		Camallanus sp. (5)	*Pleistophora* sp. + Unidentified digeneans (3)
		Presumed *Pleistophora* sp. (3)	
		Unidentified intrahepatic nematode larvae (2)	
		Unidentified intraluminal intestinal nematode (2)	Unidentified digenean + *Saprolegnia* spp. + Suggestive of renal myxosporean (1)
		Suggestive of renal myxosporean (2)	
		Presumed *Centrocestus formosanus* (1)	
		Presumed *Calyptospora* sp. (1)	*Henneguya* spp. + Unidentified digeneans (1)
		Presumed *Henneguya* spp. (1)	
		Granulomas containing remnants of nematode larvae (1)	
	Bacterial (38)	Compatible with or presumably mycobacteriosis (32)	*Mycobacterium* spp. (*Fite Faraco* positive) (18)
		Presumed columnaris diseases (6)	*Saprolegnia* spp. + Unidentified digeneans (2)
	Oomycete/Fungi (10)	Presumed *Saprolegnia* spp. (9)	*Saprolegnia* spp. + Unidentified digeneans (2)
		Pigmented fungal hypha (1)	
	Metabolic (2)	Nephrocalcinosis (1)	
		Hepatic lipidosis (1)	Chromatophoroma + *Mycobacterium* spp. (*Fite Faraco positive*) (1)
	Neoplastic (1)	Chromatophoroma (1)	
	Inconclusive (6)	Severe necrotizing and lymphohistioplasmacytic myositis (3)	
		Moderate multifocal muscle necrosis (1)	
		Mild lymphocytic and granulocytic dermatitis (1)	
		Severe granulomatous myositis, osteitis, and nephritis (1)	
	No lesions (74)		
**Facility (117)**	Parasitary (15)	Capillariids (6)	
		Presumed *Centrocestus formosanus* (2)	*Ichthyophonus* spp. + Capillariids (1)
		Presumed *Ichthyophonus* spp.(2)	
		Eustrongylides spp. (1)	
		Presumed *Piscinoodinium pillulare* (1)	
		Suggestive of amoeba (1)	
		Unidentified digeneans (1)	
		Granulomatous coelomitis with fragments of a degenerated metazoan (1)	
	Congenital (8)	Caudal peduncle malformation (8)	
	No lesions (94)		*P. pillulare* + *I. necator*
**Hobbyist (55)**	Parasitary (11)	Presumed *Piscinoodinium pillulare* (6)	
		Capillariids (2)	
		Camallanus spp. (2)	
		Presumed *Tetrahymena* spp. (1)	*Micobacterium* spp. (*Fite Faraco positive)* (3)
	Bacterial (10)	Compatible with or presumably mycobacteriosis (10)	

	Neoplastic (3)	Cromatoforoma (1)	
		Papilloma (1)	
		Sarcoma (1)	
	No lesions (31)		
**Wholesaler (22)**	Parasitary (9)	Presumed *Ichthyophthirius multifiliis* (3)	
		Presumed *Clinostomum* spp. (3)	
		Presumed *Epistylis* spp.(2)	
		Suggestive of renal myxosporidians (1)	
		Presumed *Centrocestus formosanus* (1)	*Mycobacterium* spp. (*Fite Faraco* positive) (1)
	Bacterial (4)	Compatible with or presumably mycobacteriosis (4)	Suggestive of mycobacteriosis + *C. formosanus* (1)
			Saprolegnia spp. + Unidentified digeneans + *I. multifiliis* (1)
	Oomycete (3)	Presumed *Saprolegnia* spp. (3)	
	No lesions (6)		
