# Supplementary material for: Histopathological Assessment of Diseases in Ornamental Fish from 2019 to 2023 in Minas Gerais State, Brazil
Source: Animals (Basel). 2026 Oct 9;16(20):3164. doi: 10.3390/ani16203164 (PMC13700644; doi:10.3390/ani16203164)
Supplement: Supplementary file 1 [file animals-16-03164-s001.zip › Supplementary Table S2_v2.pdf]

Table S2. Detailed summary of parasitic diseases diagnosed in the survey, indicating the main affected fish species and their respective sources

Parasitic disease	Main affected fish species (n)	Source (n)
Unidentified digeneans	*Carnegiella strigata* (15/34)	Shopkeeper (15)
	Corydoras spp.(7/34)	Shopkeeper (7)
	Colomesus asellus (3/34)	Shopkeeper (3)
Presumed *Centrocestus formosanus*	*Danio rerio* (15/32)	Farmer (13), Facilitie (2)
	Carassius auratus (8/32)	Farmer (6), Wholesaler (2)
	Xiphophorus hellerii (5/32)	Farmer (5)
	Xiphophorus maculatus (3/32)	Farmer (2), Shopkeeper (1)
Unidentified monogeneans	*Carassius auratus* (15/29)	Farmer (14), Wholesaler (1)
	Pangasianodon hypophthalmus (3/29)	Wholesaler (3)
	Poecilia reticulata (3/29)	Wholesaler (3)
Presumed *Tetrahymena* spp.	*Poecilia reticulata* (14/17)	Wholesaler (7), Farmer (6), Hobbyists (1)
Presumed *Epistylis* spp.	*Cyprinus carpio* (9/14)	Farmer (9)
Camallanus spp.	*Poecilia reticulata* (8/12)	Shopkeeper (6), Hobbyist (2)
	Pterophyllum scalare (3/12)	Shopkeeper (3)
Capillariids	*Poecilia reticulata* (6/12)	Farmer (4), Hobbyist (2)
	Danio rerio (6/12)	Facilitie (6)
Presumed *Cryptobia* spp.	*Aulonocara* spp. (7/12)	Shopkeeper (7)
	Symphysodon discus (4/12)	Farmer (4)
Presumed *Clinostomum* spp.	*Poecilia reticulata* (8/11)	Farmer (8)
	Pterophyllum scalare (3/11)	Wholesaler (3)
Presumed *Trichodina* spp.	*Carassius auratus* (9/9)	Farmer (9)
Eustrongylides spp.	*Danio rerio* (8/8)	Farmer (7), Facilitie (1)
Presumed *Ichthyophonus* spp.	*Poecilocharax weitzmani* (5/8)	Shopkeeper (5)
	Danio rerio (2/8)	Facilitie (2)
Presumed *Piscinoodinium pillulare*	*Betta splendens* (4/8)	Hobbyist (4)
	Danio rerio (3/8)	Hobbyist (2), Facilitie (1)
Presumed *Ichthyophthirius multifiliis*	*Betta splendens* (2/4)	Wholesaler (2)
	Colomesus asellus (1/4)	Wholesaler (1)
	Trichogaster trichopterus (1/4)	Wholesaler (1)
Presumed *Pleistophora* sp.	*Carnegiella strigata* (3/3)	Shopkeeper (3)
Suggestive of renal myxosporidians	*Carnegiella strigata* (1/2)	Shopkeeper (1)
	Colomesus asellus (1/2)	Shopkeeper (1)
Suggestive of amoebae	*Carassius auratus* (1/1)	Facilitie (1)
Presumed *Henneguya* spp.	*Carnegiella strigata* (1/1)	Shopkeeper (1)
Presumed *Calyptospora* spp.	*Laetacara curviceps* (1/1)	Shopkeeper (1)
